# Caveolin-1 Expression in the Dorsal Striatum Drives Methamphetamine Addiction-Like Behavior

**DOI:** 10.3390/ijms22158219

**Published:** 2021-07-30

**Authors:** Yosef Avchalumov, Alison D. Kreisler, Wulfran Trenet, Mahasweta Nayak, Brian P. Head, Juan C. Piña-Crespo, Chitra D. Mandyam

**Affiliations:** 1 Veterans Affairs San Diego Healthcare System, San Diego, CA 92161, USA; yavchalumov@vapop.ucsd.edu (Y.A.); alisonkreisler@gmail.com (A.D.K.); wtrenet@vapop.ucsd.edu (W.T.); mnayak@ucsd.edu (M.N.); bhead@ucsd.edu (B.P.H.); 2 Department of Anesthesiology, University of California San Diego, San Diego, CA 92037, USA; 3 Department of Molecular Medicine, Scripps Research, La Jolla, CA 92037, USA; jcpina-crespo@scripps.edu

**Keywords:** self-administration, caveolin, long-term potentiation, dopamine D1 receptor, cannabinoid CB1 receptor, CaMKII

## Abstract

Dopamine D1 receptor (D1R) function is regulated by membrane/lipid raft-resident protein caveolin-1 (Cav1). We examined whether altered expression of Cav1 in the dorsal striatum would affect self-administration of methamphetamine, an indirect agonist at the D1Rs. A lentiviral construct expressing Cav1 (LV-Cav1) or containing a short hairpin RNA against Cav1 (LV-shCav1) was used to overexpress or knock down Cav1 expression respectively, in the dorsal striatum. Under a fixed-ratio schedule, LV-Cav1 enhanced and LV-shCav1 reduced responding for methamphetamine in an extended access paradigm compared to LV-GFP controls. LV-Cav1 and LV-shCav1 also produced an upward and downward shift in a dose–response paradigm, generating a drug vulnerable/resistant phenotype. LV-Cav1 and LV-shCav1 did not alter responding for sucrose. Under a progressive-ratio schedule, LV-shCav1 generally reduced positive-reinforcing effects of methamphetamine and sucrose as seen by reduced breakpoints. Western blotting confirmed enhanced Cav1 expression in LV-Cav1 rats and reduced Cav1 expression in LV-shCav1 rats. Electrophysiological findings in LV-GFP rats demonstrated an absence of high-frequency stimulation (HFS)-induced long-term potentiation (LTP) in the dorsal striatum after extended access methamphetamine self-administration, indicating methamphetamine-induced occlusion of plasticity. LV-Cav1 prevented methamphetamine-induced plasticity via increasing phosphorylation of calcium calmodulin kinase II, suggesting a mechanism for addiction vulnerability. LV-shCav1 produced a marked deficit in the ability of HFS to produce LTP and, therefore, extended access methamphetamine was unable to alter striatal plasticity, indicating a mechanism for resistance to addiction-like behavior. Our results demonstrate that Cav1 expression and knockdown driven striatal plasticity assist with modulating addiction to drug and nondrug rewards, and inspire new strategies to reduce psychostimulant addiction.

## 1. Significance Statement

Methamphetamine self-administration alters expression and subcellular localization of the membrane/lipid raft protein caveolin1 (Cav1) in the dorsal striatum. We showed that lentiviral mediated overexpression of Cav1 in the dorsal striatum increases methamphetamine self-administration and enhances methamphetamine reward, and knockdown of Cav1 prevents methamphetamine addiction. We used electrophysiology to demonstrate that methamphetamine self-administration prevents HFS-induced LTP and occludes plasticity in the dorsal striatum. Cav1 overexpression altered methamphetamine-induced plasticity by increasing activity of CaMKII. Cav1 knockdown prevented HFS-induced LTP and methamphetamine-induced occlusion of plasticity. Thus, Cav1 plays a role in the synaptic effects of methamphetamine in the dorsal striatum and is necessary for the rewarding properties of methamphetamine.

## 2. Introduction

Substance use disorder is a learned behavior characterized by compulsive drug seeking that is fueled and maintained by negative affective states [1,2,3,4]. Neurobiological mechanisms in the transition from drug use to drug dependence are heavily investigated, and a relationship between dopamine and corticostriatal synaptic activity is strongly implicated [5,6]. In the dorsal striatum, dopamine D1 receptors (D1Rs) and D2 receptors (D2Rs) play a role in mediating synaptic transmission and synaptic plasticity of GABAergic medium-sized spiny neurons (MSNs; [7,8,9,10,11,12]), such that activation of these receptors produces either long-term potentiation (LTP) or long-term depression (LTD). In the context of methamphetamine exposure, protracted withdrawal from experimenter delivered methamphetamine or self-administered methamphetamine prevents high frequency stimulation (HFS)-induced LTD and produces LTP in the dorsal striatum [13,14]. However, no studies have evaluated the ongoing effect of methamphetamine addiction-like behavior on striatal plasticity, specifically, HFS-induced LTP in the dorsal striatum, and whether the alterations in striatal LTP correlate with enhancing or reducing methamphetamine addiction-like behavior. In this context, recent findings demonstrate that superfusion of methamphetamine on dorsal striatal slices reduces HFS-induced LTP, indicating methamphetamine-induced occlusion of plasticity [15,16]. More notable is that methamphetamine-induced reduction of LTP is prevented by SCH23390, indicating that D1Rs played a role in methamphetamine-induced occlusion of plasticity [15]. In addition to the dopamine system, the endocannabinoid system appears to be associated with methamphetamine and substance use disorders [9,17,18,19,20,21,22]. Furthermore, cannabinoid receptors (CBRs) in the striatum play a role in HFS-induced synaptic depression, suggesting that CBRs may be important for methamphetamine-induced occlusion of synaptic plasticity [18,19,21,22]. Given that drug-induced plasticity, especially in the dorsal striatum, plays a role in altering the reinforcing properties of drugs of abuse [21,23,24], the synaptic mechanisms in the dorsal striatum contributing to methamphetamine addiction-like behavior have yet to be investigated.

Caveolins (Cavs) are present in neuronal and non-neuronal cells in three isoforms, Cav1, 2, 3 [25]. Cav1 is a cholesterol binding protein essential for formation of membrane/lipid rafts (MLRs) in neurons, and functions as chaperones and scaffolds for assisting with signaling of several molecules, including G-protein coupled receptors [26,27]. Cav1 regulates neuronal signaling [27]. When upregulated or overexpressed in the brain, Cav1 enhances receptor function and neuronal plasticity [28,29,30]. For example, enhancing Cav1 in the brain is indicated to be a potential therapy for enhancing neuroplasticity after neurodegeneration, cerebral ischemia and stroke [29,31]. Conversely, Cav1 overexpression in non-neuronal cells has been shown to mediate tumorigenesis and enhance oxidative stress in cancer [32]. In addition, Cav1 is a target in preventing hypertrophic scarring and promoting epithelialization and wound closure [33,34]. In the context of substance use disorders, Cav1 is a target in preventing cocaine-induced vascular ischemic complications, and cocaine- and morphine-induced behavioral tolerance and structural plasticity in neuronal cells [35,36,37,38,39]. These studies indicate the bidirectional role of Cav1 in health and disease.

In the context of methamphetamine addiction-like behavior, methamphetamine self-administration followed by binge methamphetamine reduces total Cav1 expression in the dorsal striatum [40]. Furthermore, methamphetamine self-administration enhances subcellular localization of Cav1 in the MLR/buoyant fraction in the dorsal striatum [41], indicating enhanced Cav1 function. In addition to the associations between methamphetamine and Cav1, Cav1 regulates function of D1Rs [42,43], and Cav1 mediated D1R internalization is dependent on the integrity of MLRs. For example, alterations in D1R internalization by overexpression of Cav1 could play a role in cellular and behavioral tolerance via sensitized D1R function. Cannabinoid 1 receptors (CB1Rs) are also enriched in Cav1 containing domains, suggesting that alterations in Cav1 expression and activity could alter CB1R function [44]. Based on these observations, we performed genetic overexpression or knockdown of Cav1 in the dorsal striatum using a lentiviral mediated approach to determine the effects of dorsal striatal Cav1 overexpression and knockdown on methamphetamine self-administration. In parallel, we used a similar approach to determine the effects of dorsal striatal Cav1 overexpression and knockdown on sucrose self-administration. We hypothesized that dorsal striatal Cav1 overexpression would enhance methamphetamine self-administration, and that this behavior would occur in concert with enhanced synaptic plasticity in the dorsal striatum. Conversely, dorsal striatal Cav1 knockdown would reduce methamphetamine self-administration, and that this behavior would occur in concert with reduced synaptic plasticity in the dorsal striatum. We sub-hypothesized that dorsal striatal overexpression or knockdown of Cav1 would have no effects on sucrose self-administration.

## 3. Results

### 3.1. Green Fluorescent Protein (GFP) Expression Is Limited to Neurons and Is Not Observed in Astrocytes

**Figure 1:**
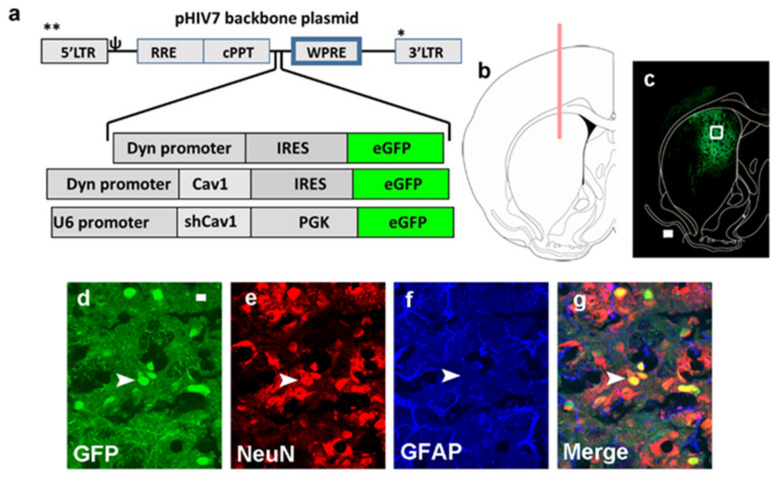
(**a**) Schematic of the lentiviral vector backbone indicating the genes of interest along with promoters that are inserted upstream of the WPRE in the pHIV-7 vector; eGFP, enhanced green fluorescent protein. **, 5’ and *, 3’ region of the backbone. (**b**) Schematic representation of a coronal section through the dorsal striatum of the adult rat brain indicating the placement of injector needle for virus infusions. (**c**) RepreScheme 2. marker for lentivirus, (**d**), NeuN (CY3, marker for neurons, (**e**)), GFAP (CY5, marker for astroglia, (**f**)), and the composite of all three images (**g**). Arrowhead points to a NeuN+GFP+ colabeled cells that do not express GFAP (**g**). Scale bar in (**c**) is 100 µm, in (**d**) is 10 µm.

Immunohistochemistry was performed to determine whether GFP labeled cells were expressed in neurons or astrocytes via co-labeling with NeuN (a protein expressed in neurons) and GFAP (a protein expressed in astrocytes). Confocal imaging demonstrated co-labeling of GFP+ cells with NeuN+ cells in the dorsal striatum (Figure 1d–g). None of the GFP+ cells were co-labeled with GFAP (Figure 1d–g).

### 3.2. Effect of LV-Cav1 and LV-shCav1 on Methamphetamine Self-Administration

Methamphetamine self-administration behavior (fixed-ratio, FR; dose–response, DR and progressive-ratio, PR sessions) did not differ between the LV-GFP rats and those that were virus naïve. Therefore, the rats from both these groups were combined and used for analysis.

**Figure 2:**
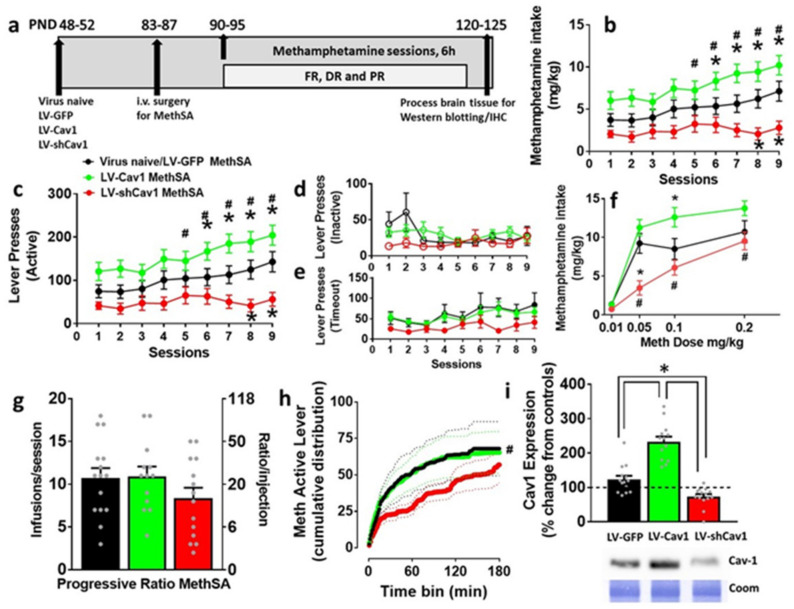
Methamphetamine self-administration in virus naïve, LV-GFP, LV-Cav1, and LV-shCav1 rats. (**a**) Schematic of the timeline of experimental design and corresponding age of rats (in postnatal days, PND) from the start to the completion of the study. FR, fixed ratio; DR, dose–response; PR, progressive ratio. (**b**) Amount of methamphetamine consumed by rats in each experimental group. (**c**) Active lever responses during extended 6 h access sessions of methamphetamine self-administration. (**d**) Inactive lever responses during extended 6 h access sessions of methamphetamine self-administration. (**e**) Active lever responses during timeout period. (**f**) Amount of methamphetamine consumed during dose response sessions. (**g**) Average number of methamphetamine deliveries earned per progressive-ratio session with corresponding number of required lever presses. (**h**) Cumulative active lever responding across progressive-ratio sessions. Solid line is the average cumulative response and dotted lines are ± SEM. (**i**) Quantitative data and qualitative blots of Cav1 in dorsal striatal tissue homogenates. Dashed line at 100% indicates expression of protein in controls. Coom, Coomassie. (**b**,**c**,**f**,**h**,**i**) Significant post hoc analysis is indicated as * *p* < 0.05 vs. LV-GFP rats and ^#^ *p* < 0.05 vs. LV-shCav1 rats. For (**b**–**d**), n = 9 virus naive; n = 10 LV-GFP, n = 17 LV-Cav1, and n = 17 LV-shCav1 rats. Some of these rats were used for electrophysiology (see Figure 6). For (**f**–**h**), n = 7 virus naive; n = 8 LV-GFP, n = 13 LV-Cav1 and n = 14 LV-shCav1 rats. For (**i**), n = 7 virus naive; n = 8 LV-GFP, n = 13 LV-Cav1, and n = 14 LV-shCav1 rats.

*FR:* we analyzed the effect of virus treatment on the amount of methamphetamine consumed. Two-way ANOVA detected a significant session × treatment interaction (F(16,400) = 3.06, *p* < 0.0001), main effect of session (F(8,400) = 15.2, *p* < 0.0001), and treatment (F(2,50) = 9.2, *p* = 0.004; Figure 2b). LV-GFP and LV-Cav1 rats increased methamphetamine intake across the nine sessions. Post hoc analysis showed that LV-Cav1 rats showed a higher drug intake compared with LV-shCav1 on days 5–9 and, compared with LV-GFP rats on days 6–9 (*p* < 0.05). LV-shCav1 rats consumed lower amount of methamphetamine compared with LV-GFP rats on days 8–9 (*p* < 0.05). When lever responses were analyzed, two-way ANOVA of active lever responses detected a significant session x treatment interaction (F(16,400) = 3.06, *p* < 0.0001), main effect of session (F(8,400) = 15.2, *p* < 0.0001), and treatment (F(2,50) = 9.2, *p* = 0.004; Figure 2c). LV-GFP and LV-Cav1 rats increased the number of active lever presses across the nine acquisition sessions. Post hoc analysis showed that LV-Cav1 rats showed a higher number of active lever presses compared with LV-shCav1 on days 5–9, and compared with LV-GFP rats on days 6–9 (*p* < 0.05). LV-shCav1 rats showed lower number of active presses compared with LV-GFP rats on days 8–9 (*p* < 0.05). Two-way ANOVA of inactive lever responses did not detect a significant session x treatment interaction, main effect of session, or treatment (Figure 2d). Two-way ANOVA of timeout lever responses did not detect a significant session × treatment interaction, or a significant main effect of treatment; however, detected a main effect of sessions (F(8,400) = 3.5, *p* = 0.0006; Figure 2e).

*Dose*–*response*: an inverted U-shaped dose–response curve is produced when animals self-administer methamphetamine on FR schedules, spanning lower doses for maintaining self-administration, and higher doses where the dose prolongs the duration of drug effects, resulting in fewer lever responses over time [45]. Dose–response curves were determined for methamphetamine self-administration across four doses (Supplementary Figures S1 and S2f). Two-way ANOVA of active lever responses are presented in Supplementary Figure S1. Two-way ANOVA of dose-intake data showed that there was a significant treatment X dose interaction (F(6,117) = 7.1, *p* < 0.0001), main effect of dose (F(3,117) = 136.9, *p* < 0.0001), and main effect of treatment (F(2,39) = 7.4, *p* = 0.001; Figure 2f). Post hoc analysis reflected that LV-shCav1 rats consumed less methamphetamine than LV-Cav1 rats at 0.05, 0.1, and 0.2 mg/kg (*p* < 0.05). Similarly, post hoc analysis showed that LV-shCav1 rats consumed less methamphetamine than LV-GFP rats at 0.05 dose of meth (*p* < 0.05), but did not differ at the other doses tested. LV-GFP rats consumed less methamphetamine than LV-Cav1 rats at 0.1 and 0.2 mg/kg doses (*p* < 0.05).

*PR:* LV-shCav1 and LV-Cav1 rats did not significantly differ from LV-GFP rats in infusions earned (*p* > 0.05 by one-way ANOVA; Figure 2g). Further analysis of active lever responses revealed significant group differences in the cumulative reinforced responses in which the LV-GFP (Kolmogorov–Smirnov D = 0.62, *p* < 0.001), and LV-Cav1 rats (Kolmogorov–Smirnov D = 0.59, *p* < 0.001) exhibited a steeper rise in lever responding than LV-shCav1 rats (Figure 2h).

### 3.3. Effect of LV-Cav1 and LV-shCav1 on Sucrose Self-Administration

**Figure 3:**
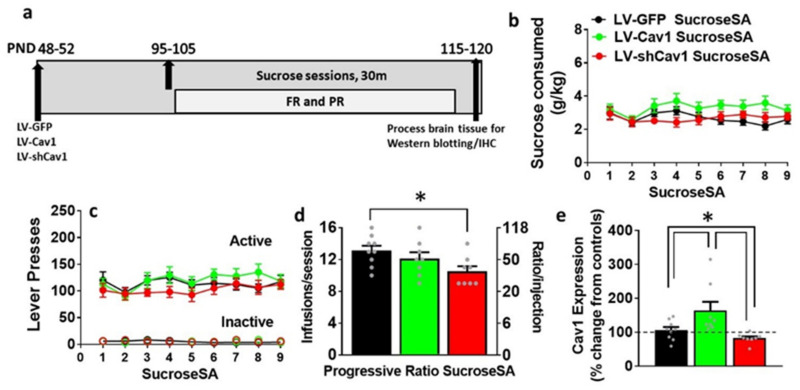
(**a**) Experimental timeline indicating the time of lentiviral expression, 30 min sucrose self-administration sessions and brain tissue collection. Corresponding age of rats from the start to the completion of the study in PND; FR, fixed ratio; PR, progressive ratio. (**b**) Amount of sucrose consumed during 30 min sucrose self-administration sessions. (**c**) Active and inactive lever responses during 30 min sucrose self-administration sessions. (**d**) Average number of sucrose deliveries earned per progressive-ratio session with corresponding number of required lever presses. Significant post hoc analysis is indicated as * *p* < 0.05. (**e**) Quantitative data and qualitative blots of Cav1 in dorsal striatal tissue homogenates. Dashed line at 100% indicates expression of protein in controls. Significant post hoc analysis is indicated as * *p* < 0.05. LV-GFP (n = 9), LV-Cav1 (n = 8), and LV-shCav1 (n = 8).

*FR*: two-way ANOVA of sucrose consumed detected a significant session x virus treatment interaction (F(16,176) = 2.0, *p* = 0.02), and main effect of session (F(8,176) = 3.4, *p* = 0.01) without a main effect of treatment (F(2,22) = 1.9, *p* = 0.1; Figure 3b). Post hoc analysis did not reveal any significant differences in sucrose consumed between groups. Two-way ANOVA of active lever responses detected a significant session x treatment interaction (F(16,176) = 1.7, *p* = 0.04), and main effect of session (F(8,176) = 4.6, *p* = 0.0001) without a main effect of treatment (F(2,22) = 0.76, *p* = 0.4; Figure 3c). Post hoc analysis did not reveal any significant differences in sucrose responses between groups. Two-way ANOVA of inactive lever responses did not detect a significant session x treatment interaction, main effect of session or treatment (Figure 3c).

*PR:* LV-shCav1 differed significantly from LV-Cav1 and LV-GFP rats in infusions earned (F(2,22) = 3.6, *p* = 0.04 by one-way ANOVA; Figure 3d). Post hoc analysis showed reduced number of infusions earned in LV-shCav1 rats compared with LV-GFP rats (*p* = 0.01). Further analysis of active lever responses revealed similar group differences in the cumulative reinforced responses (Supplementary Figure S2).

### 3.4. LV-Cav1 Enhances and LV-shCav1 Reduces Expression of Cav1 in Dorsal Striatum Tissue Homogenates

*Methamphetamine rats*: treatment effects by one-way ANOVA reflected that Cav1 expression was significantly altered in methamphetamine rats (F(3,42) = 38.1; *p* < 0.001). Post hoc analyses revealed that LV-Cav1 rats had higher levels of Cav1 compared to all groups (*p* < 0.05), and LV-shCav1 rats had lower levels of Cav1 compared with all other groups (*p* < 0.05; Figure 2i).

*Sucrose rats:* treatment effects by one-way ANOVA reflected that Cav1 expression was significantly altered in sucrose rats (F(3,26) = 5.3; *p* = 0.005). Post hoc analyses revealed that LV-Cav1 rats had higher levels of Cav1 compared to all groups (*p* < 0.05), and LV-shCav1 rats had lower levels of Cav1 compared with all other groups (*p* < 0.05; Figure 3e).

### 3.5. Basal Synaptic Transmission and Synaptic Plasticity Is Compromised after Acute Methamphetamine Treatment, but Restored in the Presence of SCH23390 and AM251 in Behavior, Virus and Drug Naive Rats

**Figure 4:**
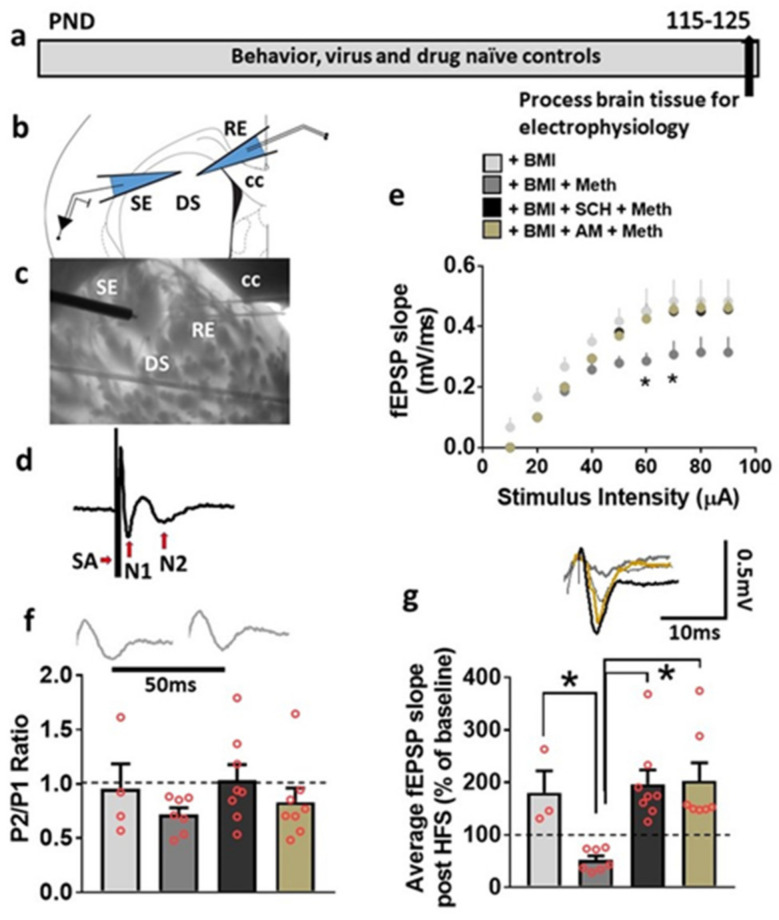
Basal synaptic transmission is reduced by superfusion of methamphetamine in the dorsal striatum in control rats. (**a**) Timeline of experimental design. (**b**) Schematic of a coronal slice representing the area of the dorsomedial striatum used for recordings with stimulation (SE) and recording electrodes (RE). (**c**) Infrared photomicrograph of a 440 µm thick corticostriatal slice from one adult male rat indicating the location of the SE and RE in the slice. Thick arrows point to the electrodes. CC: corpus callosum; DS: dorsal striatum. (**d**) Example trace of baseline population spike or fEPSP evoked in the DS. Traces are composed of a stimulus artifact (SA), non-synaptic N1 component, and a synaptically mediated N2 component. Only N2 components were analyzed for all traces. (**e**) Input/output (I/O) curve obtained by plotting the slope of fEPSPs as a function of the stimulation intensity (from 10 to 90 µA) in the dorsomedial striatum. Significant post hoc analysis is indicated as * *p* < 0.05. (**f**) Paired-pulse ratio recorded from one interstimulus interval at 50 ms. Dashed line at 1 indicates no facilitation or depression, and values greater than 1 indicate facilitation and values lower than 1 indicate depression. Inset shows an example N2 component of trace used to calculate PPR. (**g**) Average fEPSP slope of each experimental group post HFS (40 min). Dashed line at 100% indicates baseline. Inset shows a representative fEPSP waveform from BMI (gray trace), methamphetamine (dark gray trace), SCH23390 + methamphetamine (black trace), and AM + methamphetamine (brown trace) treated slices. Only N2 component of the traces are indicated for clarity. Significant post hoc analysis is indicated as * *p* < 0.05. Data are represented as mean ± SEM. Eight rats were used for the study. Number of slices: n = 3–4 BMI, n = 7 methamphetamine, n = 8 SCH23390 + methamphetamine and n = 7–8 AM + methamphetamine.

First, basal synaptic transmission was evaluated under control (BMI, 1 µM), methamphetamine (30 µM), methamphetamine + SCH23390 (10 µM), and methamphetamine + AM251 (2 µM) conditions. Repeated measures two-way ANOVA with stimulus intensity and superfusion of methamphetamine ± drugs (treatment) as independent variables and population spike or field excitatory post synaptic potential (fEPSP) slope as dependent variable detected a significant treatment x stimulus intensity interaction (F(24,198) = 1.7, *p* = 0.02), main effect of stimulus intensity (F (8,198) = 112.8, *p* < 0.0001) and main effect of treatment, (F(3,198) = 29.7, *p* < 0.0001; Figure 4e). Post hoc analysis revealed that the input/output (I/O) curve was dramatically reduced in methamphetamine treated slices compared to control, SCH23390 and AM251 treated slices, indicating that the excitability was restored in methamphetamine treated slices in the presence of SCH23390 and AM251.

Second, short-term synaptic plasticity was assessed by measuring paired pulse ratio (PPR). PPR was calculated as the change in slope of the second fEPSP relative to that of the first fEPSP after an interstimulus interval of 50 milliseconds. One-way ANOVA did not detect any treatment effects (F(3,23) = 1.1, *p* = 0.3; Figure 4f).

Lastly, under both control and methamphetamine + drugs conditions, evoked fEPSPs showed significant LTP post HFS (Figure 4g). We report the degree of LTP for each experimental group, as the average of the fEPSP slope for 40 min period of post-HFS recording. One-way ANOVA detected a significant effect of treatment (F(3,21) = 7.1, *p* = 0.001; Figure 4g). Post hoc analysis revealed significantly higher average fEPSP slope in control and methamphetamine + SCH23390 and + AM251 conditions compared with methamphetamine only condition (*p* < 0.05). Thus, these findings demonstrate that methamphetamine reduced synaptic plasticity in the dorsal striatum and SCH23390 and AM251 prevented this effect.

### 3.6. LV-GFP and LV-Cav1 Do Not Alter Synaptic Transmission and Plasticity; LV-shCav1 Produces In Vivo Deficits in Synaptic Plasticity without Altering Synaptic Transmission in Rats That Did Not Self-Administer Methamphetamine

**Figure 5:**
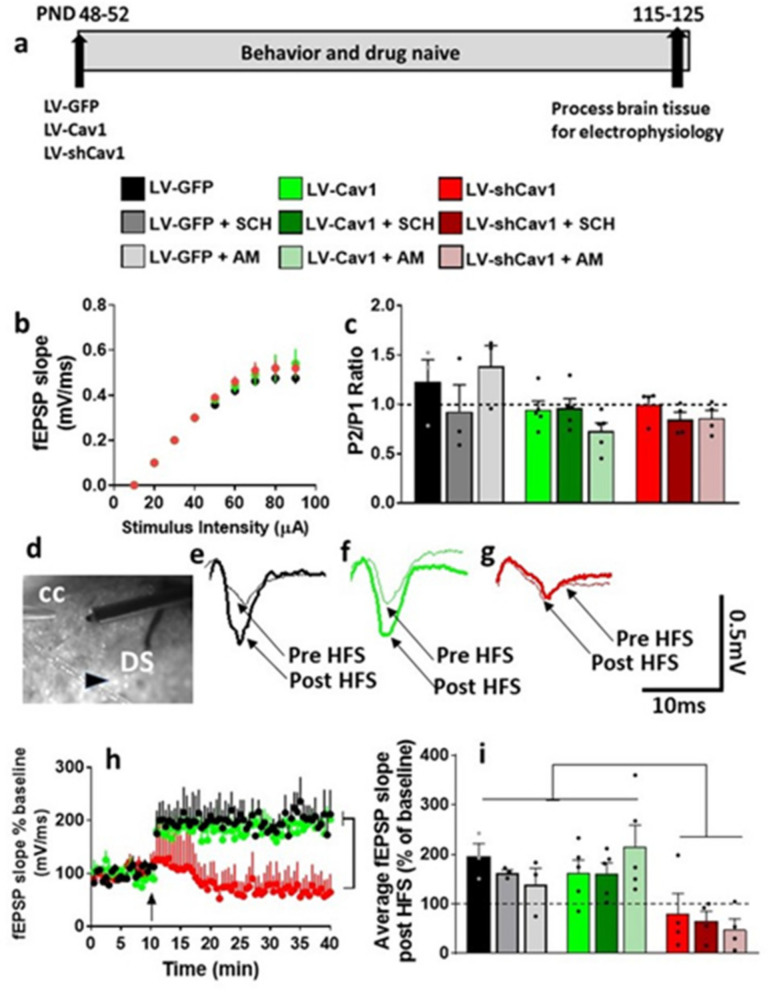
LV-GFP, LV-Cav1, and LV-shCav1 in drug and behavior naïve rats do not alter basal synaptic transmission and paired-pulse ratio, however, distinctly alter synaptic plasticity in the dorsal striatum. (**a**) Schematic of virus groups and experimental timeline of virus injections and brain tissue collection for electrophysiology. (**b**) Input/output (I/O) curve obtained by plotting the slope of fEPSPs as a function of the stimulation intensity (from 10 to 90 µA) in the dorsomedial striatum under BMI condition. (**c**) PPR recorded from one interstimulus interval at 50 ms. Dashed line at 1 indicates no facilitation or depression, and values greater than 1 indicate facilitation and values lower than 1 indicate depression. (**d**) Fluorescent photomicrograph of a 440 µm thick corticostriatal slice from one LV-GFP rat indicating the location of the stimulating and recording electrodes on the slice. Arrowhead points to a fluorescent cell. CC: corpus callosum; DS: dorsal striatum. (**e**–**g**) Representative fEPSP waveform from LV-GFP (black traces), LV-Cav1 (green traces), LV-shCav1 (red traces) slices indicating pre and post HFS traces. Only N2 component of the traces are indicated for clarity. (**h**) x–y graph of time course of fEPSPs before and after HFS in all groups under vehicle (BMI) condition. Arrow in (**h**) at 10 min points to the time of HFS. (**i**) Average fEPSP slope of each experimental group post HFS (40 min). Dashed line at 100% indicates baseline. Number of slices/rats: n = 3/3 LV-GFP, n = 5/5 LV-Cav1, n = 4/4 LV-shCav1. Significance, *p* < 0.05 of main effect is indicated in (**i**).

We then determined whether basal synaptic transmission, PPR and synaptic plasticity in the dorsal striatum were altered in LV-Cav1 and LV-shCav1 rats compared with LV-GFP rats. Striatal slices from each virus group were evaluated under control (BMI), SCH23390 and AM251 conditions. Repeated measures two-way ANOVA with stimulus intensity and virus treatment as independent variables and fEPSP slope as dependent variable did not detect a treatment x stimulus intensity interaction or main effect of treatment, however, detected a main effect of stimulus intensity under BMI (F(8,81) = 185.4, *p* < 0.0001; Figure 5b), SCH23390 (F(8,81) = 238.2, *p* < 0.0001; Supplementary Figure S3) and AM251 (F(8,81) = 177.8, *p* < 0.0001; Supplementary Figure S3) conditions. Two-way ANOVA did not detect any differences in PPR (Figure 5c). 

Under BMI, in slices from LV-GFP and LV-Cav1 rats, evoked fEPSPs showed significant LTP post HFS (Figure 5e,f,h,i). However, under BMI, in slices from LV-shCav1 rats, HFS was unable to produce LTP (Figure 5g–i). Two-way ANOVA with virus treatment and time after HFS as independent variables and fEPSP slope as dependent variable detected a significant treatment x time interaction (F(158, 632) = 2.4, *p* < 0.0001), a main effect of time (F(79,632) = 4.2, *p* < 0.0001), and a strong trend towards main effect of treatment (F(2,8) = 3.9, *p* = 0.06; Figure 5h). Post hoc analyses revealed higher average fEPSP slope in LV-GFP and LV-Cav1 slices compared with LV-shCav1 slices (*p* < 0.05).

We also report the degree of LTP for each virus group under BMI, SCH23390 and AM251 conditions, as the average of the fEPSP slope for 40 min period of post-HFS recording (Figure 5i). This was done to further investigate the mechanisms underlying LTP in each group. Two-way ANOVA detected a significant effect of virus treatment (F(2,27) = 13.2, *p* < 0.0001). Thus, these findings demonstrate that LV-shCav1 reduced synaptic plasticity in the dorsal striatum and these effects were independent of D1R and CB1R activity.

### 3.7. HFS-Induced LTP Is Differentially Altered by Methamphetamine Self-Administration in LV-GFP vs. LV-Cav1 and LV-shCav1 Rats

**Figure 6:**
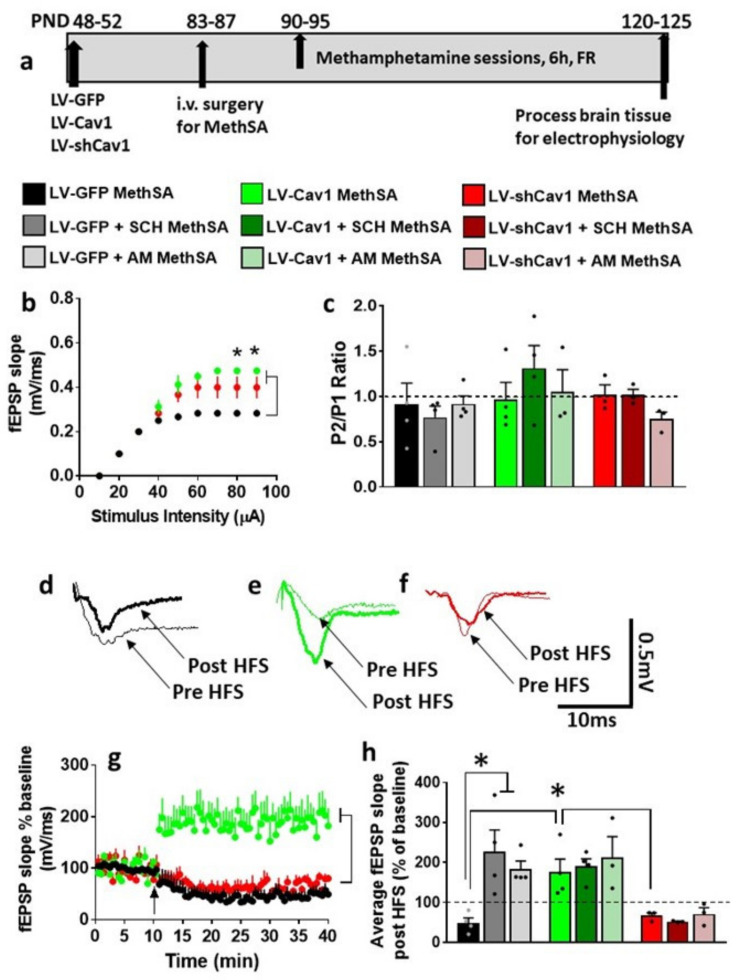
Methamphetamine self-administration in LV-GFP, LV-Cav1, and LV-shCav1 rats differentially alters basal synaptic transmission and synaptic plasticity without altering paired-pulse ratio in the dorsal striatum. (**a**) Schematic of virus groups and experimental timeline of virus injections and brain tissue collection for electrophysiology. The self-administration behavior of these rats are reported in Figure 2. (**b**) Input/output (I/O) curve obtained by plotting the slope of fEPSPs as a function of the stimulation intensity (from 10 to 90 µA) in the dorsomedial striatum under BMI condition. Significant post hoc analysis is indicated as * *p* < 0.05. (**c**) PPR recorded from one interstimulus interval at 50 ms. Dashed line at 1 indicates no facilitation or depression, and values greater than 1 indicate facilitation and values lower than 1 indicate depression. (**d**–**f**) Representative fEPSP waveform from LV-GFP (black traces), LV-Cav1 (green traces), LV-shCav1 (red traces) slices indicating pre and post HFS traces. Only N2 component of the traces are indicated for clarity. (**g**) x–y graph of time course of fEPSPs before and after HFS in all groups under vehicle (BMI) condition. Arrow in (g) at 10 min points to the time of HFS. Significance of main effect is indicated. (**h**) Average fEPSP slope of each experimental group post HFS (40 min). Dashed line at 100% indicates baseline. Significant post hoc analysis is indicated as * *p* < 0.05. Number of slices/rats: n = 4/4 LV-GFP, n = 4/4 LV-Cav1, n = 3/3 LV-shCav1.

We also determined whether basal synaptic transmission, PPR, and synaptic plasticity in the dorsal striatum were altered in LV-Cav1 and LV-shCav1 methamphetamine rats compared with LV-GFP methamphetamine rats. Striatal slices from each virus group were evaluated under control (BMI), SCH23390, and AM251 conditions. Repeated measures two-way ANOVA of I/O curves with stimulus intensity and virus treatment as independent variables and fEPSP slope as dependent variable were examined under BMI, SCH23390 and AM251 conditions: under BMI—a treatment x stimulus intensity interaction (F(16,56) = 6.6, *p* < 0.0001; Figure 6b), main effect of treatment (F(2,7) = 10.1, *p* = 0.008), and a main effect of stimulus intensity (F(8,56) = 178.6, *p* < 0.0001) was detected; under SCH23390—a treatment x stimulus intensity interaction or main effect of treatment was not evident; however, a main effect of stimulus intensity (F(8,56) = 366.2, *p* < 0.0001; Supplementary Figure S4) was detected; under AM251—a treatment x stimulus intensity interaction or main effect of treatment was not evident; however, a main effect of stimulus intensity (F(8,56) = 144, *p* < 0.0001; Supplementary Figure S4) was detected. Two-way ANOVA did not detect any differences in PPR (Figure 6c). 

Under BMI, in LV-GFP and LV-shCav1 slices, evoked fEPSPs failed to show LTP post HFS (Figure 6d,f,g,h). However, under BMI, in LV-Cav1 slices, HFS was able to produce significant LTP (Figure 6e,g–h). Two-way ANOVA with virus treatment and time after HFS as independent variables and fEPSP slope as dependent variable detected a significant treatment x time interaction (F(158, 632) = 5.4, *p* < 0.0001) and a main effect of treatment (F(2, 8) = 13.9, *p* = 0.002) without a main effect of time (F(79, 632) = 0.6, *p* = 0.9; Figure 6g). Post hoc analyses revealed lower average fEPSP slope in LV-GFP and LV-shCav1 slices compared with LV-Cav1 slices (*p* < 0.05).

We report the degree of LTP for each virus group under BMI, SCH23390, and AM251 conditions, as the average of the fEPSP slope for 40 min period of post-HFS recording (Figure 6h). This was done to further investigate the underlying LTP mechanisms in each group. Two-way ANOVA detected a significant virus treatment x blockers interaction (F(4,23) = 3.0, *p* = 0.03), main effect of virus treatment (F(2,23) = 12.6, *p* = 0.0002), and main effect of blockers (F(2,23) = 3.6, *p* = 0.04). Post hoc analyses revealed higher LTP in LV-Cav1 slices compared with LV-GFP and LV-shCav1 slices under BMI (*p* < 0.05); and higher LTP in LV-GFP slices under SCH23390 and AM251 conditions compared with BMI (*p* < 0.05). Thus, these findings demonstrate that methamphetamine self-administration distinctly produces alterations in basal synaptic transmission and synaptic plasticity in LV-GFP, LV-Cav1 and LV-shCav1 rats, and these effects were differentially altered by activity of D1R and CB1Rs.

### 3.8. Methamphetamine Self-Administration in LV-Cav1 Enhances and in LV-shCav1 Does Not Alter Phosphorylation of CaMKII in Dorsal Striatum Tissue Homogenates Compared with LV-GFP Rats

**Figure 7:**
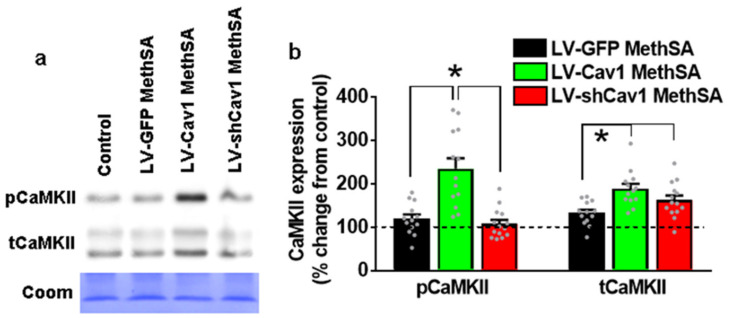
Methamphetamine self-administration in LV-Cav1 rats increases pCaMKII in the dorsal striatum. (**a**) Representative immunoblots of pCaMKII and tCaMKII used for western blotting analysis from saline control and LV-GFP, LV-Cav1, and LV-shCav1 rats that self-administered methamphetamine. Corresponding Coomassie staining (Coom) of the membrane is shown as loading control. (**b**) Quantitative analysis of protein expression in tissue from methamphetamine-exposed rats represented as percent change from saline controls. Data from control rats is indicated as dashed line at 100%. Significant post hoc analysis is indicated as * *p* < 0.05. n = 5 controls, n = 13–14 LV-GFP/virus naïve MethSA, n = 13 LV-Cav1 MethSA, n = 15 LV-shCav1 MethSA.

Previous studies have demonstrated an association of Cav1 with CaMKII, with the N-terminal domain of CaMKII harboring lipid raft targeting signals. More notable is that lipid raft targeting of CaMKII promoted activation of CaMKII at its autophosphorylation site to enhance postsynaptic signal processing at the postsynaptic density [46,47]. We therefore determined whether methamphetamine self-administration altered CaMKII expression and activation at its autophosphorylation site in LV-GFP, LV-Cav1, and LV-shCav1 rats. Western blot analyses of phopho (p)CaMKII and total (t)CaMKII were conducted on dorsal striatal tissue lysates from methamphetamine LV-GFP, LV-Cav1, and LV-shCav1 rats to determine whether enhanced LTP in LV-Cav1 rats could be attributed to changes in CaMKII activity. In LV-Cav1 rats, one-way ANOVA revealed enhanced expression of pCaMKII at the autophosphorylation site compared with LV-GFP and LV-shCav1 rats (F(3,42) = 15.7; *p* < 0.0001; Figure 7a,b). Post hoc analyses revealed higher expression of pCaMKII in LV-Cav1 rats compared with LV-GFP and LV-shCav1 rats (*p* = 0.02). Furthermore, one-way ANOVA revealed enhanced expression of tCaMKII in LV-Cav1 and LV-shCav1 rats compared with LV-GFP rats (F(3,42) = 9.8; *p* < 0.0001). Post hoc analyses revealed higher expression of tCaMKII in LV-Cav1 and LV-shCav1 rats compared with LV-GFP rats (*p* = 0.04).

## 4. Discussion

Accumulating evidence indicates that addictive drugs hijack the striatal memory systems to produce the behavioral effects [24]. Accordingly, methamphetamine exposure has been shown to alter synaptic plasticity in multiple neuronal systems of the reward circuitry, including the striatum [15,48,49,50,51,52,53]. Synaptic adaptations in the dorsal striatum underlie habit learning [54] and escalation of methamphetamine self-administration-induced altered plasticity in the dorsal striatum might therefore play a role in enhancing vulnerability to methamphetamine addiction [19]. 

The current study provided evidence that extended access methamphetamine self-administration is associated with escalation of methamphetamine intake and altered corticostriatal synaptic transmission and plasticity. In particular, while HFS produced LTP in striatal slices from methamphetamine naïve Cav1 intact (LV-GFP) rats, HFS failed to induce LTP in striatal slices from Cav1 intact rats that self-administered methamphetamine. These results indicated methamphetamine self-administration-induced blockade of LTP or occlusion of plasticity. The reduction or prevention of HFS-induced LTP by methamphetamine coincided with escalation of methamphetamine self-administration in Cav1 intact rats. Mechanistic findings demonstrated that methamphetamine self-administration-induced reduced synaptic transmission and blockade of LTP was prevented by D1R and CB1R antagonists, indicating a direct role for D1Rs and CB1Rs in methamphetamine driven occlusion of plasticity. The present study also evaluated the role of Cav1 in striatal plasticity and escalation of methamphetamine self-administration. For example, overexpression of Cav1 did not alter basal synaptic transmission and synaptic plasticity in the dorsal striatum under methamphetamine naïve conditions, however promoted tolerance to methamphetamine self-administration-induced effects on synaptic transmission and plasticity. In parallel, overexpression of Cav1 promoted a tolerance to the rewarding effects of methamphetamine, evident as increased self-administration of methamphetamine and a vertical shift in self-administration dose response function. Cellular mechanisms revealed that overexpression of Cav1 enhanced activity of CaMKII at its autophosphorylation site. Most importantly, our study showed that knockdown of Cav1 in the dorsal striatum prevented HFS-induced LTP under methamphetamine naïve and methamphetamine self-administration conditions without altering basal synaptic transmission. Knockdown of Cav1 reduced methamphetamine self-administration and prevented escalation of drug intake. Together, these observations support the conclusion that Cav1 plays a role in the synaptic effects of methamphetamine in the dorsal striatum and is necessary for the rewarding properties of methamphetamine, and represents a part of the neuronal adaptations mediating addiction.

There is general agreement that psychostimulants impair cognitive processes such as memory and learning. In addition, it is conceptualized that these behavioral impairments play a role in assisting with drug seeking behaviors and can be characterized as weakening of behavioral flexibility [55]. Certain attributes of LTP in the striatum make it an attractive model for memory processes at the synaptic level, and investigation of the effects of extended access methamphetamine self-administration on dorsal striatal LTP may provide important insights into mechanisms underlying drug-induced plasticity [19,24]. Glutamatergic neurons from several areas, including cortex, hippocampus, amygdala, and thalamus project to the dorsal striatum (corticostriatal glutamatergic system) and innervate striatal MSNs to maintain LTP [56,57]. The nigrostriatal dopaminergic system is implicated in action control, learning, and stimulus-response habits [58], and regulates the transition from controlled to compulsive drug intake, which is characterized by escalation in rodents and binging in human subjects [59]. A large body of work has focused on the contribution of the nigrostriatal system in the dorsal striatum to addiction-like behaviors via modulation of corticostriatal plasticity [60]. In support of this, drug-induced alterations in the function of the nigrostriatal and corticostriatal circuits have been hypothesized to underlie substance use disorders [21,61]. To add to these findings, the current study investigated the synaptic mechanisms in the dorsal striatum contributing to escalation of methamphetamine self-administration-induced plasticity. For example, systemic antagonism of D1Rs reduces amphetamine addiction-related behaviors including reward, self-administration and priming-induced drug seeking [62,63,64,65,66,67]. Microinjection of D1R antagonist into the dorsal striatum prevents methamphetamine-induced loss of dopamine transporters, reduces methamphetamine-induced hyperlocomotion, and some aspects of amphetamine-induced timing impulsivity, and methamphetamine reinforcement [15,68,69,70]. In addition, previous findings indicate that D1R antagonism prevented acute methamphetamine-induced reduction of HFS-LTP in the dorsal striatum [15], and combined with the current findings, it can be confirmed that D1Rs in the dorsal striatum played a role in the behavioral and synaptic effects that supported escalation of methamphetamine intake. In addition, synaptic transmission and plasticity in dorsal striatal synapses are modulated by CB1Rs [71]. Furthermore, systemic or striatal antagonism of CB1Rs reduced multiple methamphetamine addiction-related behaviors including reward, self-administration and priming-induced drug seeking [72,73,74,75,76,77,78]. To add to these findings, our results supported a role for CB1Rs in the dorsal striatum in methamphetamine-induced synaptic effects that were associated with escalation of methamphetamine intake.

In this study we also describe the effect of enhancing and reducing levels of Cav1 in the dorsal striatum on methamphetamine addiction-like behaviors. LV-Cav1 enhanced Cav1 protein expression by ~100% and LV-shCav1 reduced expression by ~20%. A striking observation was that LV-Cav1 rats demonstrated a higher rate of responding during extended access sessions and had an upward shift in the dose response curve compared with Cav1 intact rats. This behavioral profile is associated with increased LTP in the dorsal striatum without concomitant changes in synaptic transmission and PPR. These results suggest that neuroadaptive changes in LV-Cav1 rats produced by escalated methamphetamine intake are most evident when there is high demand on synaptic function, such as during HFS. Most notable is that the alterations in synaptic plasticity occurred in concert with increased activity of CaMKII in the dorsal striatum. Increased activity of CaMKII at its autophosphorylation site could be facilitating the enhanced synaptic plasticity observed in LV-Cav1 rats and promoting the rewarding effects of methamphetamine and assisting with drug vulnerable phenotype [79,80]. Another important finding is that LV-shCav1 rats did not develop methamphetamine addiction-like behavior with extended access to the drug. This was evident as a lack of escalation in drug intake and downward shift in dose response curve. LV-shCav1 showed reduced propensity to lever-press for methamphetamine in PR sessions, indicating reduced motivation to seek methamphetamine. This drug resistant behavioral profile was associated with a lack of HFS-induced LTP in the dorsal striatum under reduced Cav1 expression condition, albeit intact synaptic transmission and short-term plasticity. These results suggest that Cav1 is required for methamphetamine-induced aberrant synaptic plasticity to strengthen distinct drug associated cues and enhance drug reward, and facilitates the higher order plasticity that is needed for drug addiction [39].

Synaptic plasticity in the dorsal striatum is heterogeneous, in that LTD is predominantly observed in the dorsolateral striatum and LTP is observed in the dorsomedial striatum after HFS [81]. Concurrently, behavioral studies show that dorsomedial and dorsolateral striatum may have distinct roles in learning and memory functions dependent on the dorsal striatum [82]. Furthermore, addiction related studies show that the dorsomedial striatum is associated with goal-directed actions, whereas, the dorsolateral striatum is associated with habitual actions [20]. The current findings evaluated the role of Cav1 in the dorsal striatum in methamphetamine addiction-like behaviors. Therefore, a potential limitation in the interpretation of our findings is that the role of Cav1 in the dorsolateral vs. dorsomedial striatum were not investigated and such distinctions may offer new roles of Cav1 in addictive disorders.

Lentiviral mediated gene therapy is a commonly used tool in behavioral neuroscience [83]. LV-Cav1 and LV-shCav1 animals were tested for methamphetamine self-administration and sucrose self-administration. Rats that self-administered sucrose were considered behavioral control groups, since sucrose is a non-drug reinforcer commonly used to determine the specificity of behavioral effects. Accordingly, operant self-administration of sucrose was not altered under FR sessions in LV-Cav1 and LV-shCav1 conditions, and we propose that the sucrose groups represent the pure effect of overexpression or knockdown of Cav1 to be compared with the effect of these manipulations with methamphetamine self-administration. However, our results indicate that LV-shCav1 rats have a reduced propensity to lever-press for sucrose reward in PR sessions. Therefore, LV-shCav1 rats generally showed reduced motivation to seek sucrose or methamphetamine. However, the lack of an effect on FR responding for sucrose could be due to the different paradigm of reinforcement schedule used for sucrose (30 min) vs. methamphetamine (6 h), and that the effects of LV-Cav1 and LV-shCav1 could be different if sucrose sessions were performed under extended access. Another potential limitation in the interpretation of these findings is that the current lentiviral strategy with LV-shCav1 may have non-specific effects, including infecting neurons of multiple phenotypes in the dorsal striatum, which could have assisted with differential effects on FR and PR responding for sucrose. An important future pursuit would be to use the validated transgenic rats expressing Cre recombinase in either D1-MSNs or D2-MSNs, and Cre-dependent knockdown of Cav1 in these neuronal populations to dissect the role of Cav1, in these specific neuronal populations, in methamphetamine, and sucrose behaviors [84]. Nevertheless, to our knowledge, our study is the first to use the lentiviral approach to specifically enhance or reduce Cav1 expression in the dorsal striatum in order to study its role in methamphetamine addiction. In summary, our findings suggest that Cav1 promotes brain reactivity in the context of methamphetamine addiction. Specifically, Cav1 may be assisting with enhanced neuronal integrity via higher order plasticity in the dorsal striatum. Therefore, Cav1 may be a promising therapeutic target to treat methamphetamine use disorders. 

## 5. Methods

### 5.1. Animals

Experimental procedures were carried out in strict adherence to the NIH Guide for the Care and Use of Laboratory Animals and approved by the Institutional Animal Care and Use Committee of VA San Diego Healthcare System. One hundred and four adult male rats on a Long Evans background (bred at the VA Vivarium or purchased from Charles River laboratories), weighing 200–250 g and 7 weeks old at the start of the experiment, were housed two per cage in a temperature-controlled vivarium under a reverse light/dark cycle (lights off 8:00 a.m.–8:00 p.m.), and completed the study. Food and water were provided ad libitum.

### 5.2. Viral Vector Construction, Surgery and Viral Gene Transfer

Lentivirus expressing GFP, Cav1 ((LV)-Cav1; LV-Cav1) and short hairpin RNA to Cav1 (shRNA; (LV-shCav1)) was generated at the UCSD viral vector core facility using published methods ([85]; Figure 1).

*HIV1*-*Dyn*-*Cav1*: to link the D1-MSN specific dynorphin (Dyn) promoter (generous gift from Dr. Martin Darvas, University of Washington; [86]), a BamH1-EcoR1 DNA fragment containing the Dyn promoter was inserted into the BamH1-EcoR1 sites of peGFP-N1 (Clontech, Mountain View, CA, USA). The resulting plasmid was designated pDyn-eGFP. To link the Dyn promoter with Cav1 cDNA, a 685bp Cav1 cDNA isolated from the pCRII-TOPO vector (Invitrogen, Waltham, MA, USA) was used to generate the pDyn-Cav1. The Dyn-eGFP or Dyn-Cav1 cassette was then cloned into the BamHI site of the HIV1 vector backbone plasmid pHIV7 [87] and the resulting plasmid was designated pHIV1-Dyn-eGFP or pHIV1-Dyn-Cav1.

*HIV1-shCav1-PGK-Puro*: shRNAs targeting the rat Cav1 was selected and corresponding sense and antisense oligonucleotides were synthesized, annealed, and cloned downstream of the mouse U6 promoter in the pSiren-RetroQ plasmid (Clontech, Mountain View, CA, USA). The insertion was confirmed by sequencing. The sequence of the sense strand DNA oligomer of the shCav1 was as follows: 5′ GATCC GGAAATGTGATCGTGGTGCAA CTTCCTGTCA TTGTACCATGATCATATTTCCTTTTTG 3′. The puromycin-resistant gene (Puro) driven by the phosphoglycerate kinase (PGK) promoter contained in the pSiren-RetroQ was replaced with the eGFP gene. The U6-shCav1-PGK-eGFP cassette was then cloned into the BamHI site of the HIV1 vector backbone plasmid pHIV7 [87] and the resulting plasmid was designated pHIV1-shCav1-PGK-eGFP.

*Production of HIV1 based lentivirus vectors*: Lentivirus vectors were produced by transient co-transfection of 293T cells maintained in Dulbecco’s Modified Eagle Medium (DMEM) with 10% FCS. 293T cells in 150 mm dishes were co-transfected by polyethylenimine (PEI) method with each HIV1 vector plasmid, pLP1 and pLP2 (Invitrogen, Waltham, MA, USA), and pCMV-G [88]. Conditioned media at day 1, 2, and 3 post transfection were collected, filtered through a 0.45 µm filter, and concentrated by centrifugation at 7000 rpm for 16 h at 4 °C with a Sorvall GS-3 rotor. The resulting pellets were resuspended with buffer containing 10 mM Tris HCl, pH 7.8, 1 mM MgCl2, and 3% sucrose.

*Titering of HIV1 vectors by real-time qPCR*: HIV1-CMV-GFP vector (1 × 10^9^ iu/mL) was used as the standard. HEK293 cells in a 6-well plate were infected with different amounts of viruses in the presence of polybrene (4 µg/mL). Infected cells were passaged once every 4 days and cell DNAs were prepared at day 14 post infection by the DNeasy Blood & Tissue kit (Qiagen Science, MD, USA). Real-time Q-PCR was performed using a primer set selected from the WPRE sequence in the HIV1 vector backbone. Viral titer ~10^11^ viral particle/μL was used.

*Stereotactic injections*: eighty-two rats (7 weeks old) were anesthetized with 2–4% isoflurane mixed with oxygen. Using standard stereotaxic procedures [89], 30-gauge stainless steel injectors were placed above targeted brain region. Stereotaxic bilateral infusions of LV-GFP, LV-Cav1 or LV-shCav1 were applied to the DS (AP, 0.2 mm from bregma; ML, ±2.3 mm from bregma; DV, −4.5, −5.1, and −5.4 from dura) with a stainless steel injector attached to a syringe pump connected by plastic tubing (Figure 1e,f). Infusions occurred at a flow rate of 1 µL/min with a total volume of 10 µL (3.5 µL infused at −4.5 mm, 4.5 µL infused at −5.1 mm, and 2 µL infused at −5.4 mm) per side. The injector was left in place an additional 5 min to minimize diffusion up the injector tract. Immediately after surgery, Flunixin^®^ (2.5 mg/kg, s.c.) was given as analgesic, and Cefazolin was administered as antibiotic. For all experiments, accuracy of injection coordinates was confirmed by visualization of GFP-expressing cells in 60 µm tissue sections (Figure 1). Four weeks following LV injections, rats either underwent surgery for jugular vein catheter implantation (for methamphetamine self-administration) or did not undergo any additional surgery (intended for sucrose self-administration/drinking).

### 5.3. Intravenous Methamphetamine Self-Administration

*Intravenous catheterization surgery*: fifty-three rats (LV-GFP (n = 10), LV-Cav1 (n = 17), LV-shCav1 (n = 17) and virus naïve (n = 9)) underwent surgery for catheter implantation for intravenous self-administration 4–5 weeks after virus injections. Surgery, catheter maintenance and catheter patency were performed according to our previous publications [90,91].

*Training and maintenance on the extended access schedule (days 1–9)*: one week after i.v. surgeries, all rats were trained to press a lever according to fixed-ratio 1 (FR1) schedule of methamphetamine reinforcement (0.05 mg/kg/injection of methamphetamine (provided by NIDA)) in operant boxes (Med Associates, Fairfax, VT, USA) under extended access conditions (6 h access per day). FR data were analyzed as number of reinforced or non-reinforced (active or inactive) lever presses per session.

*Dose–response (days 10–13)*: following nine sessions, animals underwent dose–response studies in which the methamphetamine dose administered was changed each day: 0.01, 0.05, 0.1, and 0.2 mg/kg/injection (within-subjects). Dose–response sessions were maintained at 6 h and FR1 schedule.

*Progressive ratio (day 14)*: the following day after dose response sessions, responding was reinforced on a progressive ratio (PR) schedule of reinforcement [92]. On this schedule, the number of responses required for reinforcement incremented progressively, and each session continued until a breakpoint (defined as the number of infusions obtained before 1 h elapsed with no infusions) was reached. For example, in the PR schedule, the response requirement began at 1 response/injection and increased according to the following equation: responses/injection = (5 × e(injection number × 0.2)) − 5. When a rat failed to achieve the response requirement within 1 h, the session ended. Sessions were capped at 6 h; however, no PR session exceeded 3 h. Breakpoints were determined for 0.05 mg/kg/injection.

### 5.4. Oral Sucrose Self-Administration

*Training and maintenance on the limited access schedule* (*days 1*–*9*): twenty-five rats (LV-GFP (n = 9), LV-Cav1 (n = 8), LV-shCav1 (n = 8)) were then trained to orally self-administer sucrose solution (10% *w*/*v*, 30 min sessions, under FR1 schedule; Figure 3a). Before sucrose sessions began, animals received two training sessions that were conducted overnight in the operant chambers, and active lever presses were rewarded with tap water (100 µL per reinforced response). This allowed us to determine whether the animal learned to (1) press the correct lever for a fluid reward and (2) drink the administered fluid from the delivery/sipper cup. All animals distinguished the active versus the inactive lever and consumed tap water dispensed during the water training sessions (data not shown). During 30 min sucrose sessions, 100 µL of 10% sucrose was delivered following an active lever press (similar to methamphetamine sessions, a response on the active lever resulted in a delivery into a sipper cup, followed by a 20 s time-out period). Each delivery was paired for 1 s with white stimulus light over the active lever. Response during the time-out or on the inactive lever was recorded but resulted in no programmed consequences). 

*Progressive ratio (day 10)*: the following day after the last FR session, responding was reinforced on a PR schedule of reinforcement. PR schedule for sucrose was similar to the schedule used for methamphetamine, with the reward changed to 10% sucrose. Breakpoints were determined for 10% sucrose.

### 5.5. Brain Tissue Collection

All rats were euthanized via rapid decapitation under light isoflurane anesthesia (3–5%) and brain tissue was either processed for immunohistochemistry, western blotting, or electrophysiology. A cohort of methamphetamine and all sucrose rats were euthanized 1 h after conclusion of the PR session (for western blotting and immunohistochemistry). The left hemisphere was snap frozen for western blotting and the right hemisphere was post-fixed in 4% paraformaldehyde for immunohistochemistry. Another cohort of methamphetamine rats were euthanized 14–16 h after an FR1 session and brain tissue were processed for electrophysiological analysis. Brain tissue of virus injected behavior naïve rats and a group of age-matched behavior/virus naïve were processed for electrophysiological analysis.

### 5.6. Validation of Virus Infection

Post fixed tissue containing the right hemisphere was sliced in 60 μm sections along the coronal plane in a cryostat. A section from the injection site of the dorsal striatum was mounted on Superfrost^®^ Plus slides and dried overnight, and was visualized for expression of GFP under a fluorescent microscope (AxioImager A2). Examination of striatal sections demonstrated GFP labeling confined to the dorsal striatum in all the rats (Figure 1c). Immunohistochemistry was performed according to our previous publication to determine the phenotype of GFP labeled cells [40]. Sections were labeled with GFP (1:500; rabbit polyclonal; ab290; Abcam, Cambridge, MA, USA), antibodies against astrocytes (GFAP; 1:500, chicken polyclonal, cat# ab4674; Abcam) and Neurons (NeuN; mouse monoclonal, 1:50, cat# MAB377; EMD Millipore, Temecula, CA, USA). Confocal microscope was used to determine co-labeling. Virus expression was confirmed under fluorescence before slices were used for ex vivo electrophysiology (Figure 5d).

### 5.7. Western Blotting

Tissue punches from 300 μm thick sections of dorsal striatum from the left hemisphere around the injector needle track were collected from methamphetamine groups (virus naïve and LV-GFP (n = 15), LV-Cav1 (n = 13), LV-shCav1 (n = 14)), sucrose groups (LV-GFP (n = 9), LV-Cav1 (n = 8), LV-shCav1 (n = 8)) encompassing the injection site were homogenized in a refrigerated bead mill homogenizer (Next Advance) in buffer (320 mM sucrose, 5 mM HEPES, 1 mM EGTA, 1 mM EDTA, 1% SDS, with Protease Inhibitor Cocktail and Phosphatase Inhibitor Cocktails II and III diluted 1:100; Sigma, St. Louis, MO, USA), heated at 95 degrees C for five minutes, and stored at −80 degrees C until determination of protein concentration by a detergent-compatible Lowry method (Bio-Rad, Hercules, CA, USA). Tissue from a separate group of age-matched, saline self-administration rats (controls, (n = 5)) were used as control drug/sucrose naïve condition in western blotting analyses. Behavior data for these rats have been previously published [15]. Samples were mixed (1:1) with a Laemmli sample buffer containing β-mercaptoethanol before electrophoresis. Samples (20 μg per lane) were run on 10% SDS-PAGE gels (Bio-Rad) and transferred to polyvinylidene fluoride membranes (PVDF pore size 0.2 μm). Membranes were blocked with 5% milk (*w*/*v*) in TBST (25 mM Tris–HCl (pH 7.4), 150 mM NaCl, and 0.1% Tween 20 (*v*/*v*)) for 2–4 h at room temperature and were incubated with the primary antibody for 16–20 h at 4 °C: antibody for Cav1 (1:500, cat# D46G3, molecular weight 22 kDa; Cell Signaling, Danvers, MA, USA); phosphorylated (p) CamKII Tyr-286 (rabbit polyclonal, 1:200, Abcam cat# ab5683, molecular weight 50 kDa); total (t) CaMKII (rabbit polyclonal, 1:200, Abcam cat# ab52476, molecular weight 47 kDa). Membranes were then washed with TBST and incubated for 1 h at room temperature with horseradish peroxide-conjugated secondary antibody (1:1000). Following subsequent washes, immunoreactivity was detected using SuperSignalWest Dura chemiluminescence detection reagent (Thermo Scientific, Waltham, MA) and images were collected using a digital imaging system (Azure Imager c600). For normalization purposes, membranes were incubated with 0.125% Coomassie stain for 5 min and washed three times for 5–10 min in destain solution. Densitometry was performed using ImageJ software (NIH). The signal value of the band of interest following subtraction of the background calculation was then expressed as a ratio of the corresponding Coomassie signal (following background subtraction). This ratio of expression for total protein was then expressed as a percent of the control sample included on the same blot. For analysis of phosphorylated proteins, the ratio of expression of phosphorylated protein to the total protein was first calculated and then expressed as a percent of the control sample included on the same blot.

### 5.8. Slice Preparation for Electrophysiology and Electrophysiological Analysis

Behavior naïve LV-GFP (n = 3), LV-Cav1 (n = 5), and LV-shCav1 (n = 4) and methamphetamine self-administered LV-GFP (n = 4), LV-Cav1 (n = 4), and LV-shCav1 (n = 3) and virus and behavior naïve (n = 8) rats were anesthetized with isoflurane and killed by rapid decapitation. Brains were quickly removed and placed in ice-cold artificial cerebrospinal fluid (ACSF) containing (in mM): 125 NaCI, 26 NaHCO_3_, 4 KCI, 1.25 NaHPO_4_, 2 CaCl_2_, 1 MgCl_2_, and 10 glucose bubbled with 95% oxygen and 5% CO_2_ [15]. Brains were trimmed on the dorsal side at an angle of approximately 140° from the horizontal plane and glued to a vibratome base (Leica VT1000S). Thick slices (440 μm) containing the dorsal striatum were obtained and used for recordings. Three-four slices were transferred to a submerged chamber and incubated with oxygenated ACSF at 25 °C for at least 1–1.5 h before initiating recordings. Recordings were made in the dorsal striatum (Figure 4b,c), in a submersion-type recording chamber superfused with oxygenated ACSF at a rate of 2–3 mL/min at 25 °C and positioned on the stage of an upright motorized microscope (Olympus BX51 WI, Scientifica, Clarksburg, NJ, USA) equipped with a back Illuminated sCMOS camera (Prime 95B, Photometrics, Tucson, AZ, USA) and a broad-spectrum LED illuminator (pE-300, CoolLED).

### 5.9. Field Potential Recordings

To study basal synaptic transmission, paired-pulse ratio (PPR), and HFS-induced LTP, local field potentials were recorded in acute brain slices in the presence of GABA_A_ receptor antagonist bicuculline methiodide (BMI, 1 µM; Sigma; [93]).

A group of rats (n = 8) that were methamphetamine and virus naïve were used as drug and virus naïve controls to determine whether acute methamphetamine exposure altered HFS-induced LTP and whether acute methamphetamine-induced synaptic plasticity was dependent on D1R and CB1R in the dorsal striatum. In these rats, slices were recorded under control (ACSF) conditions, after superfusion of 30 µM methamphetamine in ACSF [15], after superfusion of 10 µM SCH23390 in ACSF (Sigma) + 30 µM methamphetamine [15], or after superfusion of 2 µM AM251 in ACSF containing bovine serum albumin (0.5 g/L) (Sigma, [94]) + 30 µM methamphetamine. Slices were continuously super-perfused with drugs for at least 30 min before the start of each recording session and until the end of the experiment.

A group of methamphetamine naïve rats either expressing LV-GFP (n = 3), LV-Cav1 (n = 5), or LV-shCav1 (n = 4) were used as behavior naïve controls to determine the effect of LV-GFP, LV-Cav1 and LV-shCav1 on HFS-induced LTP and whether virus-induced synaptic plasticity was dependent on D1R and CB1R in the dorsal striatum. Rats that self-administered methamphetamine either expressing LV-GFP (n = 4), LV-Cav1 (n = 4) or LV-shCav1 (n = 3) were used to determine the effect of methamphetamine self-administration in LV-GFP, LV-Cav1, and LV-shCav1 rats on HFS-induced LTP and whether treatment-driven synaptic plasticity was dependent on D1R and CB1R in the dorsal striatum. Slices were continuously super-perfused with drugs for at least 30 min before the start of each recording session and until the end of the experiment.

Population spikes or field excitatory postsynaptic potentials (fEPSPs) were evoked by extracellular stimulation (0.03 Hz, 0.2 milliseconds) in the dorsal striatum using a silver-coated tungsten wire stimulating electrode (50 μm, A-M System; Figure 4c) placed in the dorsomedial striatum in line with the recording electrode. fEPSPs were recorded using ACSF-filled patch pipettes with tip resistances of 2–4 MΩ. Pipettes were pulled from borosilicate glass capillaries (PG150T-10, Harvard Apparatus, Holliston, MA, USA) using a micropipette puller (PC-10, Narishige, Amityville, NY, USA). At least three slices per rat were used for recordings. 

Basal synaptic transmission was analyzed by generating stimulus/response curves or input/output (I/O) curves prior to each synaptic plasticity experiment. I/O curves were generated by plotting stimulus intensity (10–90 µA) versus fEPSP slope [95]. Traces were composed of a stimulus artifact, nonsynaptic N1 component and a synaptically mediated N2 component [96]. Only N2 components were analyzed for all traces. The slope of fEPSPs (N2 component) were measured from the initial 2 to 5 milliseconds of the rising phase to about half-peak time of the synaptic response. For the remainder of the experiment, the test stimulus intensity was set to elicit a fEPSP that is approximately 40–50% of the maximum response recorded during the I/O measurements. Paired-pulse ratios (P2/P1) were evaluated by dividing the slope of second fEPSP by the slope of first fEPSP obtained at 50 millisecond inter-stimulus interval. fEPSPs at this constant test stimulus intensity were monitored for a period of 25 min to ensure stable responses before induction of LTP. 

For induction of synaptic plasticity or LTP in the dorsal striatum, the following high-frequency stimulation HFS stimulation paradigm was used: four 1 s, 100 Hz trains delivered 10 s apart [97]. For comparisons of treatment effects on fEPSP slope between slices, values for each recording were normalized to the average slope for the 10 min of baseline before HFS was initiated. Data was acquired, filtered (highpass, 0.1 Hz; lowpass 3 kHz), and amplified a using a computer-controlled patch-clamp amplifier (MultiClamp 700B, Molecular Devices, San Jose, CA, USA) and digitized using an analog to digital converter (Digidata 1550A1, Molecular Devices). Analysis of fEPSP slope was performed using pClamp10.4 software (Molecular Devices).

### 5.10. Statistical Analyses

The methamphetamine and sucrose self-administration data are expressed as the average total number of lever responses per session. Methamphetamine or sucrose self-administration during the 6 h FR maintenance and dose–response sessions was examined using a two-way repeated-measures analysis of variance (ANOVA). For maintenance sessions, active and inactive lever presses were analyzed separately. One-way ANOVAs were used to determine group differences in total methamphetamine/sucrose deliveries earned per PR session. A one-way ANOVA was conducted to determine differences between drug and behavior naïve controls, LV-GFP, LV-Cav1, and LV-shCav1 rats for western blots of all proteins followed by post hoc test for each protein. Western blot analysis was conducted on percent change values from age-matched naive controls. Effects on paired-pulse ratio and effects of treatment groups or treatment conditions on fEPSP slope were analyzed using a one-way or two-way ANOVA. Post hoc analyses were conducted with Sidak’s multiple comparisons test. Significance was set at *p* < 0.05. Data were analyzed using GraphPad Prism version 7. Data are expressed as mean ± SEM in all graphs.

## Data Availability

Data will be made available upon reasonable request to the corresponding author.

## Supplementary Materials

The following are available online at https://www.mdpi.com/article/10.3390/ijms22158219/s1.

## References

[1] Redish A.D. (2004). Addiction as a computational process gone awry. Science.

[2] Koob G.F. (1999). Stress, corticotropin-releasing factor, and drug addiction. Ann. N. Y. Acad. Sci..

[3] Koob G.F. (2009). Neurobiological substrates for the dark side of compulsivity in addiction. Neuropharmacology.

[4] Koob G.F. (2013). Addiction is a Reward Deficit and Stress Surfeit Disorder. Front. Psychiatry.

[5] Vanderschuren L.J., Kalivas P.W. (2000). Alterations in dopaminergic and glutamatergic transmission in the induction and expression of behavioral sensitization: A critical review of preclinical studies. Psychopharmacology.

[6] Koob G.F., Volkow N.D. (2016). Neurobiology of addiction: A neurocircuitry analysis. Lancet Psychiatry.

[7] Calabresi P., Maj R., Mercuri N.B., Bernardi G. (1992). Coactivation of D1 and D2 dopamine receptors is required for long-term synaptic depression in the striatum. Neurosci. Lett..

[8] Calabresi P., Maj R., Pisani A., Mercuri N.B., Bernardi G. (1992). Long-term synaptic depression in the striatum: Physiological and pharmacological characterization. J. Neurosci..

[9] Surmeier D.J., Plotkin J., Shen W. (2009). Dopamine and synaptic plasticity in dorsal striatal circuits controlling action selection. Curr. Opin. Neurobiol..

[10] Shen W., Flajolet M., Greengard P., Surmeier D.J. (2008). Dichotomous dopaminergic control of striatal synaptic plasticity. Science.

[11] Pawlak V., Kerr J.N. (2008). Dopamine receptor activation is required for corticostriatal spike-timing-dependent plasticity. J. Neurosci..

[12] Avchalumov Y., Mandyam C.D. (2020). Synaptic Plasticity and its Modulation by Alcohol. Brain Plast..

[13] Moriguchi S., Nishi M., Sasaki Y., Takeshima H., Fukunaga K. (2015). Aberrant behavioral sensitization by methamphetamine in junctophilin-deficient mice. Mol. Neurobiol..

[14] Huang X., Chen Y.Y., Shen Y., Cao X., Li A., Liu Q., Li Z., Zhang L.B., Dai W., Tan T. (2017). Methamphetamine abuse impairs motor cortical plasticity and function. Mol. Psychiatry.

[15] Avchalumov Y., Trenet W., Piña-Crespo J., Mandyam C. (2020). SCH23390 Reduces Methamphetamine Self-Administration and Prevents Methamphetamine-Induced Striatal LTD. Int. J. Mol. Sci..

[16] Abraham W.C. (2008). Metaplasticity: Tuning synapses and networks for plasticity. Nat. Rev. Neurosci..

[17] Guerin A.A., Nestler E.J., Berk M., Lawrence A.J., Rossell S.L., Kim J.H. (2021). Genetics of methamphetamine use disorder: A systematic review and meta-analyses of gene association studies. Neurosci. Biobehav. Rev..

[18] Centonze D., Battista N., Rossi S., Mercuri N.B., Finazzi-Agrò A., Bernardi G., Calabresi P., Maccarrone M. (2004). A critical interaction between dopamine D2 receptors and endocannabinoids mediates the effects of cocaine on striatal gabaergic Transmission. Neuropsychopharmacology.

[19] Gerdeman G.L., Partridge J.G., Lupica C.R., Lovinger D.M. (2003). It could be habit forming: Drugs of abuse and striatal synaptic plasticity. Trends Neurosci..

[20] Lipton D.M., Gonzales B.J., Citri A. (2019). Dorsal Striatal Circuits for Habits, Compulsions and Addictions. Front. Syst. Neurosci..

[21] Mathur B.N., Lovinger D.M. (2012). Endocannabinoid-dopamine interactions in striatal synaptic plasticity. Front. Pharmacol..

[22] Thomas M.J., Beurrier C., Bonci A., Malenka R.C. (2001). Long-term depression in the nucleus accumbens: A neural correlate of behavioral sensitization to cocaine. Nat. Neurosci..

[23] Chiamulera C., Piva A., Abraham W.C. (2021). Glutamate receptors and metaplasticity in addiction. Curr. Opin. Pharm..

[24] Neuhofer D., Kalivas P. (2018). Metaplasticity at the addicted tetrapartite synapse: A common denominator of drug induced adaptations and potential treatment target for addiction. Neurobiol. Learn. Mem..

[25] Williams T.M., Lisanti M.P. (2004). The Caveolin genes: From cell biology to medicine. Ann. Med..

[26] Allen J.A., Halverson-Tamboli R.A., Rasenick M.M. (2007). Lipid raft microdomains and neurotransmitter signalling. Nat. Rev. Neurosci..

[27] Stary C.M., Tsutsumi Y.M., Patel P.M., Head B.P., Patel H.H., Roth D.M. (2012). Caveolins: Targeting pro-survival signaling in the heart and brain. Front. Physiol..

[28] Egawa J., Zemljic-Harpf A., Mandyam C.D., Niesman I.R., Lysenko L.V., Kleschevnikov A.M., Roth D.M., Patel H.H., Patel P.M., Head B.P. (2018). Neuron-Targeted Caveolin-1 Promotes Ultrastructural and Functional Hippocampal Synaptic Plasticity. Cereb. Cortex.

[29] Zhong W., Huang Q., Zeng L., Hu Z., Tang X. (2019). Caveolin-1 and MLRs: A potential target for neuronal growth and neuroplasticity after ischemic stroke. Int. J. Med. Sci..

[30] Mandyam C.D., Schilling J.M., Cui W., Egawa J., Niesman I.R., Kellerhals S.E., Staples M.C., Busija A.R., Risbrough V.B., Posadas E. (2017). Neuron-Targeted Caveolin-1 Improves Molecular Signaling, Plasticity, and Behavior Dependent on the Hippocampus in Adult and Aged Mice. Biol. Psychiatry.

[31] Wang S., Leem J.S., Podvin S., Hook V., Kleschevnikov N., Savchenko P., Dhanani M., Zhou K., Kelly I.C., Zhang T. (2021). Synapsin-caveolin-1 gene therapy preserves neuronal and synaptic morphology and prevents neurodegeneration in a mouse model of AD. Mol. Ther. Methods Clin. Dev..

[32] Wang S., Wang N., Zheng Y., Zhang J., Zhang F., Wang Z. (2017). Caveolin-1: An Oxidative Stress-Related Target for Cancer Prevention. Oxid. Med. Cell Longev..

[33] Jozic I., Sawaya A.P., Pastar I., Head C.R., Wong L.L., Glinos G.D., Wikramanayake T.C., Brem H., Kirsner R.S., Tomic-Canic M. (2019). Pharmacological and Genetic Inhibition of Caveolin-1 Promotes Epithelialization and Wound Closure. Mol. Ther..

[34] Kruglikov I.L., Scherer P.E. (2019). Caveolin-1 as a target in prevention and treatment of hypertrophic scarring. NPJ Regen. Med..

[35] Sáez C.G., Pereira-Flores K., Ebensperger R., Panes O., Massardo T., Hidalgo P., Mezzano D., Pereira J. (2014). Atorvastatin reduces the proadhesive and prothrombotic endothelial cell phenotype induced by cocaine and plasma from cocaine consumers in vitro. Arterioscler. Thromb. Vasc. Biol..

[36] Cui W., Ren Y., Wang S., Zeng M., Han S., Li J., Han R. (2018). The role of caveolin-1 in morphine-induced structural plasticity in primary cultured mouse cerebral cortical neurons. Neurosci. Lett..

[37] Ujcikova H., Hlouskova M., Cechova K., Stolarova K., Roubalova L., Svoboda P. (2017). Determination of μ-, δ- and κ-opioid receptors in forebrain cortex of rats exposed to morphine for 10 days: Comparison with animals after 20 days of morphine withdrawal. PLoS ONE.

[38] Chakrabarti S., Chang A., Liu N.J., Gintzler A.R. (2016). Chronic opioid treatment augments caveolin-1 scaffolding: Relevance to stimulatory μ-opioid receptor adenylyl cyclase signaling. J. Neurochem..

[39] Eisinger K.R.T., Chapp A.D., Swanson S.P., Tam D., Lopresti N.M., Larson E.B., Thomas M.J., Lanier L.M., Mermelstein P.G. (2020). Caveolin-1 regulates medium spiny neuron structural and functional plasticity. Psychopharmacology.

[40] Somkuwar S.S., Fannon M.J., Head B.P., Mandyam C.D. (2016). Methamphetamine reduces expression of caveolin-1 in the dorsal striatum: Implication for dysregulation of neuronal function. Neuroscience.

[41] Kreisler A.D., Terranova M.J., Somkuwar S.S., Purohit D.C., Wang S., Head B.P., Mandyam C.D. (2020). In vivo reduction of striatal D1R by RNA interference alters expression of D1R signaling-related proteins and enhances methamphetamine addiction in male rats. Brain Struct. Funct..

[42] Kong M.M., Hasbi A., Mattocks M., Fan T., O’Dowd B.F., George S.R. (2007). Regulation of D1 dopamine receptor trafficking and signaling by caveolin-1. Mol. Pharm..

[43] Voulalas P.J., Schetz J., Undieh A.S. (2011). Differential subcellular distribution of rat brain dopamine receptors and subtype-specific redistribution induced by cocaine. Mol. Cell Neurosci..

[44] Bari M., Oddi S., De Simone C., Spagnolo P., Gasperi V., Battista N., Centonze D., Maccarrone M. (2008). Type-1 cannabinoid receptors colocalize with caveolin-1 in neuronal cells. Neuropharmacology.

[45] Kitamura O., Wee S., Specio S.E., Koob G.F., Pulvirenti L. (2006). Escalation of methamphetamine self-administration in rats: A dose-effect function. Psychopharmacology.

[46] Du F., Saitoh F., Tian Q.B., Miyazawa S., Endo S., Suzuki T. (2006). Mechanisms for association of Ca^2+^/calmodulin-dependent protein kinase II with lipid rafts. Biochem. Biophys. Res. Commun..

[47] Suzuki T., Du F., Tian Q.B., Zhang J., Endo S. (2008). Ca^2+^/calmodulin-dependent protein kinase IIalpha clusters are associated with stable lipid rafts and their formation traps PSD-95. J. Neurochem..

[48] Swant J., Chirwa S., Stanwood G., Khoshbouei H. (2010). Methamphetamine reduces LTP and increases baseline synaptic transmission in the CA1 region of mouse hippocampus. PLoS ONE.

[49] Chen G., Wei X., Xu X., Yu G., Yong Z., Su R., Tao L. (2020). Methamphetamine Inhibits Long-Term Memory Acquisition and Synaptic Plasticity by Evoking Endoplasmic Reticulum Stress. Front. Neurosci..

[50] Heysieattalab S., Naghdi N., Hosseinmardi N., Zarrindast M.R., Haghparast A., Khoshbouei H. (2016). Methamphetamine-induced enhancement of hippocampal long-term potentiation is modulated by NMDA and GABA receptors in the shell-accumbens. Synapse.

[51] Ishikawa A., Kadota T., Kadota K., Matsumura H., Nakamura S. (2005). Essential role of D1 but not D2 receptors in methamphetamine-induced impairment of long-term potentiation in hippocampal-prefrontal cortex pathway. Eur. J. Neurosci..

[52] Nishioku T., Shimazoe T., Yamamoto Y., Nakanishi H., Watanabe S. (1999). Expression of long-term potentiation of the striatum in methamphetamine-sensitized rats. Neurosci. Lett..

[53] Shahidi S., Komaki A., Sadeghian R., Asl S.S. (2019). Different doses of methamphetamine alter long-term potentiation, level of BDNF and neuronal apoptosis in the hippocampus of reinstated rats. J. Physiol. Sci..

[54] Jog M.S., Kubota Y., Connolly C.I., Hillegaart V., Graybiel A.M. (1999). Building neural representations of habits. Science.

[55] Keramati M., Durand A., Girardeau P., Gutkin B., Ahmed S.H. (2017). Cocaine addiction as a homeostatic reinforcement learning disorder. Psychol. Rev..

[56] Yager L.M., Garcia A.F., Wunsch A.M., Ferguson S.M. (2015). The ins and outs of the striatum: Role in drug addiction. Neuroscience.

[57] Calabresi P., Gubellini P., Centonze D., Picconi B., Bernardi G., Chergui K., Svenningsson P., Fienberg A.A., Greengard P. (2000). Dopamine and cAMP-regulated phosphoprotein 32 kDa controls both striatal long-term depression and long-term potentiation, opposing forms of synaptic plasticity. J. Neurosci..

[58] Yin H.H., Ostlund S.B., Knowlton B.J., Balleine B.W. (2005). The role of the dorsomedial striatum in instrumental conditioning. Eur. J. Neurosci..

[59] Belin D., Everitt B.J. (2008). Cocaine seeking habits depend upon dopamine-dependent serial connectivity linking the ventral with the dorsal striatum. Neuron.

[60] Everitt B.J., Robbins T.W. (2013). From the ventral to the dorsal striatum: Devolving views of their roles in drug addiction. Neurosci. Biobehav. Rev..

[61] Balleine B.W., Liljeholm M., Ostlund S.B. (2009). The integrative function of the basal ganglia in instrumental conditioning. Behav. Brain Res..

[62] Brennan K.A., Carati C., Lea R.A., Fitzmaurice P.S., Schenk S. (2009). Effect of D1-like and D2-like receptor antagonists on methamphetamine and 3,4-methylenedioxymethamphetamine self-administration in rats. Behav. Pharm..

[63] Carati C., Schenk S. (2011). Role of dopamine D1- and D2-like receptor mechanisms in drug-seeking following methamphetamine self-administration in rats. Pharm. Biochem. Behav..

[64] Bardo M.T., Valone J.M., Bevins R.A. (1999). Locomotion and conditioned place preference produced by acute intravenous amphetamine: Role of dopamine receptors and individual differences in amphetamine self-administration. Psychopharmacology.

[65] Mizoguchi H., Yamada K., Mizuno M., Mizuno T., Nitta A., Noda Y., Nabeshima T. (2004). Regulations of methamphetamine reward by extracellular signal-regulated kinase 1/2/ets-like gene-1 signaling pathway via the activation of dopamine receptors. Mol. Pharm..

[66] Gu S.M., Cha H.J., Seo S.W., Hong J.T., Yun J. (2020). Dopamine D1 receptor antagonist reduces stimulant-induced conditioned place preferences and dopamine receptor supersensitivity. Naunyn Schmiedebergs Arch. Pharm..

[67] Nguyen J.D., Aarde S.M., Cole M., Vandewater S.A., Grant Y., Taffe M.A. (2016). Locomotor Stimulant and Rewarding Effects of Inhaling Methamphetamine, MDPV, and Mephedrone via Electronic Cigarette-Type Technology. Neuropsychopharmacology.

[68] Cheng R.K., Liao R.M. (2020). Examination of the effects of SCH23390 and raclopride infused in the dorsal striatum on amphetamine-induced timing impulsivity measured on a differential reinforcement of low-rate responding (DRL) task in rats. Behav. Brain Res..

[69] Koshikawa N., Mori E., Oka K., Nomura H., Yatsushige N., Maruyama Y. (1989). Effects of SCH23390 injection into the dorsal striatum and nucleus accumbens on methamphetamine-induced gnawing and hyperlocomotion in rats. J. Nihon Univ. Sch. Dent..

[70] Gross N.B., Duncker P.C., Marshall J.F. (2011). Striatal dopamine D1 and D2 receptors: Widespread influences on methamphetamine-induced dopamine and serotonin neurotoxicity. Synapse.

[71] Gerdeman G., Lovinger D.M. (2001). CB1 cannabinoid receptor inhibits synaptic release of glutamate in rat dorsolateral striatum. J. Neurophysiol..

[72] Anggadiredja K., Nakamichi M., Hiranita T., Tanaka H., Shoyama Y., Watanabe S., Yamamoto T. (2004). Endocannabinoid system modulates relapse to methamphetamine seeking: Possible mediation by the arachidonic acid cascade. Neuropsychopharmacology.

[73] Jing L., Qiu Y., Zhang Y., Li J.X. (2014). Effects of the cannabinoid CB_1_ receptor allosteric modulator ORG 27569 on reinstatement of cocaine- and methamphetamine-seeking behavior in rats. Drug Alcohol Depend..

[74] Landa L., Sulcova A., Slais K. (2006). Involvement of cannabinoid CB1 and CB2 receptor activity in the development of behavioural sensitization to methamphetamine effects in mice. Neuro Endocrinol. Lett..

[75] Nawata Y., Yamaguchi T., Fukumori R., Yamamoto T. (2019). Inhibition of Monoacylglycerol Lipase Reduces the Reinstatement of Methamphetamine-Seeking and Anxiety-Like Behaviors in Methamphetamine Self-Administered Rats. Int. J. Neuropsychopharmacol..

[76] Rodriguez J.S., Boctor S.Y., Flores L.C., Phelix C.F., Martinez J.L. (2011). Local pretreatment with the cannabinoid CB1 receptor antagonist AM251 attenuates methamphetamine intra-accumbens self-administration. Neurosci. Lett..

[77] Yu L.L., Wang X.Y., Zhao M., Liu Y., Li Y.Q., Li F.Q., Wang X., Xue Y.X., Lu L. (2009). Effects of cannabinoid CB1 receptor antagonist rimonabant in consolidation and reconsolidation of methamphetamine reward memory in mice. Psychopharmacology.

[78] Yu L.L., Zhou S.J., Wang X.Y., Liu J.F., Xue Y.X., Jiang W., Lu L. (2011). Effects of cannabinoid CB_1_ receptor antagonist rimonabant on acquisition and reinstatement of psychostimulant reward memory in mice. Behav. Brain Res..

[79] Steinkellner T., Mus L., Eisenrauch B., Constantinescu A., Leo D., Konrad L., Rickhag M., Sorensen G., Efimova E.V., Kong E. (2014). In vivo amphetamine action is contingent on alphaCaMKII. Neuropsychopharmacology.

[80] Giese K.P., Fedorov N.B., Filipkowski R.K., Silva A.J. (1998). Autophosphorylation at Thr286 of the alpha calcium-calmodulin kinase II in LTP and learning. Science.

[81] Partridge J.G., Tang K.C., Lovinger D.M. (2000). Regional and postnatal heterogeneity of activity-dependent long-term changes in synaptic efficacy in the dorsal striatum. J. Neurophysiol..

[82] Devan B.D., White N.M. (1999). Parallel information processing in the dorsal striatum: Relation to hippocampal function. J. Neurosci..

[83] White M., Whittaker R., Gándara C., Stoll E.A. (2017). A Guide to Approaching Regulatory Considerations for Lentiviral-Mediated Gene Therapies. Hum. Gene Ther. Methods.

[84] Pardo-Garcia T.R., Garcia-Keller C., Penaloza T., Richie C.T., Pickel J., Hope B.T., Harvey B.K., Kalivas P.W., Heinsbroek J.A. (2019). Ventral Pallidum Is the Primary Target for Accumbens D1 Projections Driving Cocaine Seeking. J. Neurosci..

[85] Mastrangelo L., Kim J.E., Miyanohara A., Kang T.H., Friedmann T. (2012). Purinergic signaling in human pluripotent stem cells is regulated by the housekeeping gene encoding hypoxanthine guanine phosphoribosyltransferase. Proc. Natl. Acad. Sci. USA.

[86] Darvas M., Palmiter R.D. (2015). Specific contributions of N-methyl-D-aspartate receptors in the dorsal striatum to cognitive flexibility. Neuroscience.

[87] Yam P.Y., Li S., Wu J., Hu J., Zaia J.A., Yee J.K. (2002). Design of HIV vectors for efficient gene delivery into human hematopoietic cells. Mol. Ther..

[88] Yee J.K., Miyanohara A., LaPorte P., Bouic K., Burns J.C., Friedmann T. (1994). A general method for the generation of high-titer, pantropic retroviral vectors: Highly efficient infection of primary hepatocytes. Proc. Natl. Acad. Sci. USA.

[89] Galinato M.H., Lockner J.W., Fannon-Pavlich M.J., Sobieraj J.C., Staples M.C., Somkuwar S.S., Ghofranian A., Chaing S., Navarro A.I., Joea A. (2018). A synthetic small-molecule Isoxazole-9 protects against methamphetamine relapse. Mol. Psychiatry.

[90] Galinato M.H., Orio L., Mandyam C.D. (2015). Methamphetamine differentially affects BDNF and cell death factors in anatomically defined regions of the hippocampus. Neuroscience.

[91] Mandyam C.D., Wee S., Eisch A.J., Richardson H.N., Koob G.F. (2007). Methamphetamine self-administration and voluntary exercise have opposing effects on medial prefrontal cortex gliogenesis. J. Neurosci..

[92] Richardson N.R., Roberts D.C. (1996). Progressive ratio schedules in drug self-administration studies in rats: A method to evaluate reinforcing efficacy. J. Neurosci. Methods.

[93] Kang S., Cox C.L., Gulley J.M. (2018). High frequency stimulation-induced plasticity in the prelimbic cortex of rats emerges during adolescent development and is associated with an increase in dopamine receptor function. Neuropharmacology.

[94] Adermark L., Talani G., Lovinger D.M. (2009). Endocannabinoid-dependent plasticity at GABAergic and glutamatergic synapses in the striatum is regulated by synaptic activity. Eur. J. Neurosci..

[95] Petersen R.P., Moradpour F., Eadie B.D., Shin J.D., Kannangara T.S., Delaney K.R., Christie B.R. (2013). Electrophysiological identification of medial and lateral perforant path inputs to the dentate gyrus. Neuroscience.

[96] Misgeld U., Okada Y., Hassler R. (1979). Locally evoked potentials in slices of rat neostriatum: A tool for the investigation of intrinsic excitatory processes. Exp. Brain Res..

[97] Avchalumov Y., Piña-Crespo J.C., Woodward J.J., Mandyam C.D. (2020). Acute Ethanol Exposure Enhances Synaptic Plasticity in the Dorsal Striatum in Adult Male and Female Rats. Brain Plast..

