# Supplementary material for: Caveolin-1 Expression in the Dorsal Striatum Drives Methamphetamine Addiction-Like Behavior
Source: Int J Mol Sci. 2021 Jul 30;22(15):8219. doi: 10.3390/ijms22158219 (PMC8348638; doi:10.3390/ijms22158219)
Supplement: Supplementary file 1 [file ijms-22-08219-s001.zip › ijms-1278177-supplementary.pdf]

Caveolin-1 expression in the dorsal striatum drives methamphetamine addiction-like behavior

Yosef Avchalumov[1], Alison D. Kreisler[1], Wulfran Trenet[1], Mahasweta Nayak[1], Brian P. Head[1,2], Juan C. Piña-Crespo[3] and Chitra D. Mandyam[1,2]

Supplementary figure S1

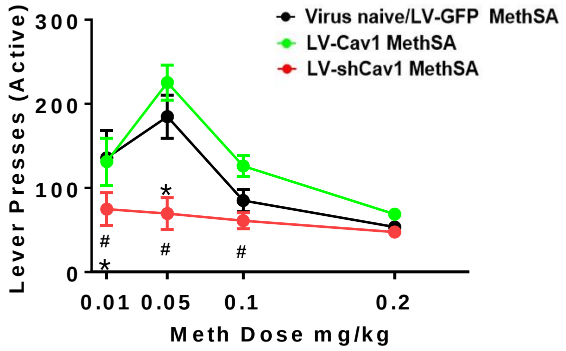

Lever responses during dose-response in methamphetamine rats: Two-way ANOVA of active lever responses showed that there was a significant treatment X dose interaction (F(6,117)=5.7, p<0.0001), main effect of dose (F(3,117)=31.9, p<0.0001) and main effect of treatment (F(2,39)=6.3, p=0.004). Posthoc analysis reflected that LV-shCav1 rats pressed the active lever fewer than LV-Cav1 rats at lower doses of meth (0.01, 0.05 and 0.1 mg/kg) but did not differ when responding for the higher dose (0.2 mg/kg). Similarly, posthoc analysis showed that LV-shCav1 rats pressed the active lever fewer than LV-GFP rats at lower doses of meth (0.01 and 0.05 mg/kg) but did not differ when responding for the higher dose (0.1 and 0.2 mg/kg).

Supplementary figure S2

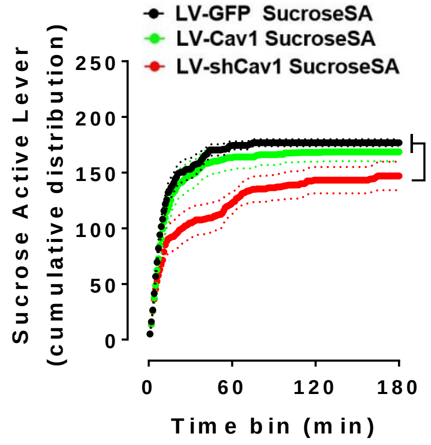

Lever responses during PR session in sucrose rats: Further analysis of active lever responses revealed significant group differences in the cumulative reinforced responses in which the LV-GFP (Kolmogorov-Smirnov D=0.88, p<0.0001), and LV-Cav1 rats (Kolmogorov-Smirnov D=0.84, p<0.0001) exhibited a steeper rise in lever responding than LV-shCav1 rats for sucrose reward.

Supplementary figure S3

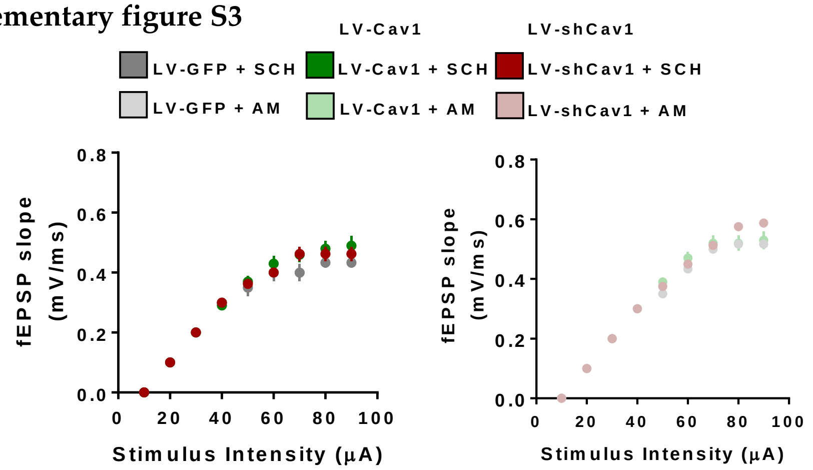

<u>*I/O curves in virus injected methamphetamine naive rats under SCH23390 and AM251 conditions:*</u> Repeated measures two-way ANOVA with stimulus intensity and virus treatment as independent variables and fEPSP slope as dependent variable did not detect a treatment x stimulus intensity interaction or main effect of treatment, however, detected a main effect of stimulus intensity under SCH23390 ($F_{(8,81)} = 238.2$, $p < 0.0001$; left panel) and AM251 ($F_{(8,81)} = 177.8$, $p < 0.0001$; right panel) conditions.

Supplementary figure S4

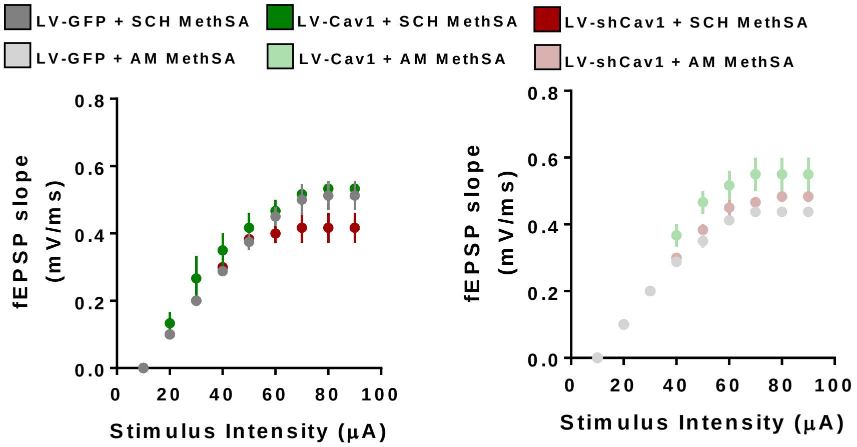

<u>*I/O curves in virus injected methamphetamine rats under SCH23390 and AM251 conditions:*</u> Repeated measures two-way ANOVA of I/O curves with stimulus intensity and virus treatment as independent variables and fEPSP slope as dependent variable were examined under SCH23390 and AM251 conditions: under SCH23390- a treatment x stimulus intensity interaction or main effect of treatment was not evident, however, a main effect of stimulus intensity ($F_{(8,56)} = 366.2$, $p < 0.0001$; left panel) was detected; under AM251- a treatment x stimulus intensity interaction or main effect of treatment was not evident, however, a main effect of stimulus intensity ($F_{(8,56)} = 144$, $p < 0.0001$; right panel) was detected.
